# Synthesis, antioxidant and cytoprotective activity evaluation of C-3 substituted indole derivatives

**DOI:** 10.1038/s41598-021-94904-z

**Published:** 2021-07-29

**Authors:** Beata Jasiewicz, Weronika Kozanecka-Okupnik, Michał Przygodzki, Beata Warżajtis, Urszula Rychlewska, Tomasz Pospieszny, Lucyna Mrówczyńska

**Affiliations:** 1 Faculty of Chemistry, Adam Mickiewicz University, Uniwersytetu Poznańskiego 8, 61-614 Poznań, Poland; 2 Department of Cell Biology, Faculty of Biology, Adam Mickiewicz University, Uniwersytetu Poznańskiego 6, 61-614 Poznań, Poland

**Keywords:** Cell death, Chemical modification, Natural products, Chemical biology, Organic chemistry

## Abstract

A series of fifteen indole derivatives substituted at the C-3 position were synthesized and characterized. The antioxidant activity of all derivatives was investigated by three in vitro antioxidant assays, and the derivative with pyrrolidinedithiocarbamate moiety was the most active as a radical scavenger and Fe^3+^-Fe^2+^ reducer. It can be stated that possible hydrogen and electron transfer mechanism is suggested for the quenching of the free radical. Moreover, the indolyl radical stabilization and the presence of unsubstituted indole nitrogen atom are mandatory for the observed antioxidant activity, which strongly depends on the type of the substituent directly connected to the methylene group at the C-3 position. Human red blood cells (RBC) have been used as a cell model to study derivatives interaction with the cell membrane. Haemolytic activity and RBC shape transformation were observed for certain derivatives in a concentration-dependent manner. However, most of the derivatives at sublytic concentration showed high cytoprotective activity against oxidative haemolysis induced by 2,2′-azobis(2-methylpropionamidine) dihydrochloride (AAPH). The cytoprotective properties of derivatives can be explained mostly due to their interactions with the RBC membrane components. Taking together, theoretical estimations and experimental data confirm the beneficial interactions between the selected C-3 substituted indole derivatives and the RBC membrane under oxidative stress conditions. These results encourage us to further structural optimization of C-3 substituted indole derivatives as potent antioxidant compounds.

## Introduction

Reactive oxygen species (ROS) are a natural by-product of cellular aerobic metabolism and play a significant role in many biological processes. Many exogenous sources can also induce ROS as smoke, pollutions, xenobiotics, or ionizing radiations. The increase in ROS level may lead to oxidative stress and, in effect, to damage of cellular macromolecules, including DNA, proteins, and lipids. Therefore, oxidative stress plays a role in ageing and developing many diseases, including cardiovascular, neurodegenerative, and autoimmune diseases^1^.

Antioxidants are involved in the prevention of oxidative stress and ROS-induced cellular damage. Therefore, natural compounds and their derivatives have been used for a long time to treat oxidative stress-related diseases^2^. Among natural and synthetic antioxidants, indole derivatives melatonin^3^ and serotonin and its derivatives, as 5-hydroxytryptofol, 5-methoxytryptamine, and 5-methoxytryptofol^4^, deserve special attention. Moreover, fluvastatin, the synthetic inhibitor of 3-hydroxy-3-methylglutaryl coenzyme A (HMG-CoA) reductase, with the unique structure of a mevalonolactone derivative of a fluorophenyl-substituted indole ring, exerts cytoprotective effects against oxidative stress induced by hydroxyl radicals and peroxide anions formed in the Fenton reaction^5^. Furthermore, indole-3-propionamide derivatives were found to reduce *cis-*platin induced ROS^6^ and scavenge hydroxyl radical directly^7^.

Gramine [3-(dimethylaminomethyl)indole] (**1**) is the main indole-alkaloid present in barley (*Hordeum vulgare* L.). It is characterized by broad physiological activity; therefore, it is used to synthesize new compounds with multifaceted therapeutic properties compared with starting molecule, including antibacterial, antiviral, antitumor, and antioxidant properties^8,9^. In addition, our previous studies have shown that selected gramine-uracils conjugates^10^ and gramine-triazole conjugates^11^ are much more active than gramine as cytoprotective compounds against in vitro induced oxidative damage human red blood cells (RBC).

According to Silveira et al.^12^, the substituent nature present at the C-3 position seems to significantly affect indole derivatives antioxidant properties. Thus, we were encouraged to explore the antioxidant potency of C-3 substituted indole derivatives. In this study, the synthesis, crystallographic, spectroscopic analysis, and molecular modeling of a series of new and already known indole derivatives obtained by functionalization of gramine molecule by the reaction of *N,O*-diacetyl-indole-3-carbinol with different alcohols is presented. Considering the interesting variety of biological activities, including antioxidant properties, seen in compounds containing phthalimide^13^, imidazole^14,15^, and pyrrolidinedithiocarbamate^16–18^ linkages, we decided to expand our research on the compounds having these functionalities present in a molecule.

To study the structure–activity relationships, the antioxidant potential of indole derivatives was evaluated in the cell-free assays, namely a 2,2′-diphenyl-1-picrylhydrazyl (DPPH) assay, a ferrous ion (Fe^2+^) chelating assay, and Fe^3+^-Fe^2+^ reducing power assay. Human RBC were used in the in vitro haemolysis assay to assess the possible membrane-perturbing activity of new compounds. They are widely used as a model of mammalian cell membranes in general and a haemolytic activity assay is a versatile tool for cytotoxicity assessment of any potential blood-contacting compounds^11,18,19^. To evaluate the in vitro cytoprotective activity of gramine derivatives against ROS-induced cell membrane damage, the oxidative stress-induced haemolysis assays with 2,2'-azobis(2-methylpropionamidine) dihydrochloride (AAPH) as ROS generator, was used. Since the antioxidant activity and cytoprotective properties of the compounds are significantly related to the compound structure^18,20–22^, the goal of our study was (i) to analyze the structure–activity relationships of derivatives as antioxidant and cytoprotective agents, (ii) to indicate the most effective derivatives, and (iii) to propose the molecular mechanism of their action.

## Results and discussion

### Synthesis and characterization of indole derivatives

**Figure 1:**
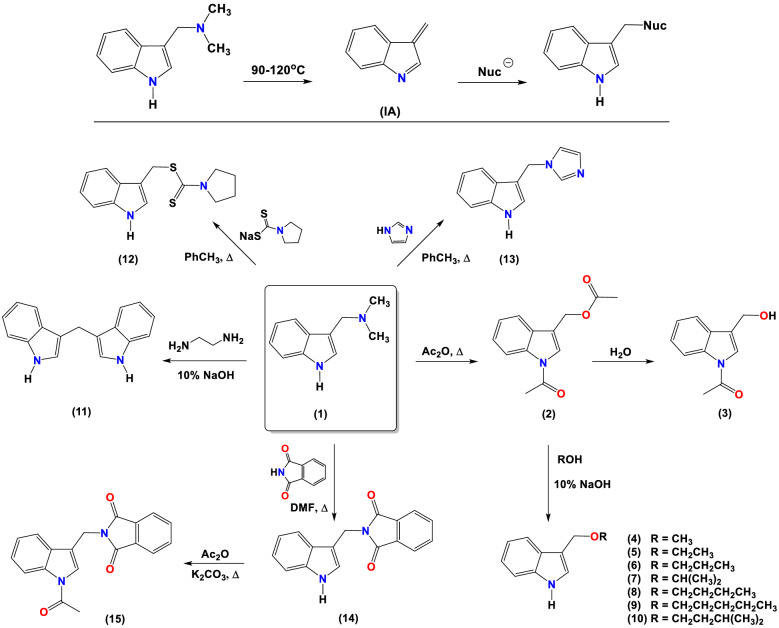
Synthesis of indole derivatives **2–15.** (ChemDraw Professional 18.0).

Indole derivatives described in this paper were obtained by nucleophilic substitution. It is known that gramine undergoes a deamination process under thermal conditions (Fig. 1)^23–25^. The intermediate product formed, alkylideneindolenine (**1A**), reacts with a nucleophile, leading to the desired product without converting the substrate into *N*-methyl iodide or *N*-oxide. The chemical modifications of the lead gramine structure were made at different points: the preparation of a series of gramine derivatives synthesized by the reaction of *N,O*-diacetylindole-3-carbinol [*N,O*-diacetyl I3C] (**2**) with different alcohols, and the synthesis of gramine derivatives containing pyrrolidinedithiocarbamate, imidazole and phthalimide linkages (Fig. 1). Compounds **2**, **4** and **5** were synthesized as previously described in the literature^26^. The same procedure was applied to the synthesis of new (**6**–**10)** gramine derivatives. Hydrolysis of *N,O*-diacetyl-indole-3-carbinol gave compound **3**. The reaction of gramine with sodium pyrrolidinedithiocarbamate, imidazole, or phthalimide gave compounds **12**, **13**, and **14**, respectively. Compounds **3**^27^, **13**^28^,**14**^29,30^ and **15**^31^ are known from the literature but obtained by a different synthesis route. Known derivative **11**^32,33^, DIM was obtained as a by-product during the reaction of gramine with ethylenediamine and 10% NaOH.

**Figure 2:**
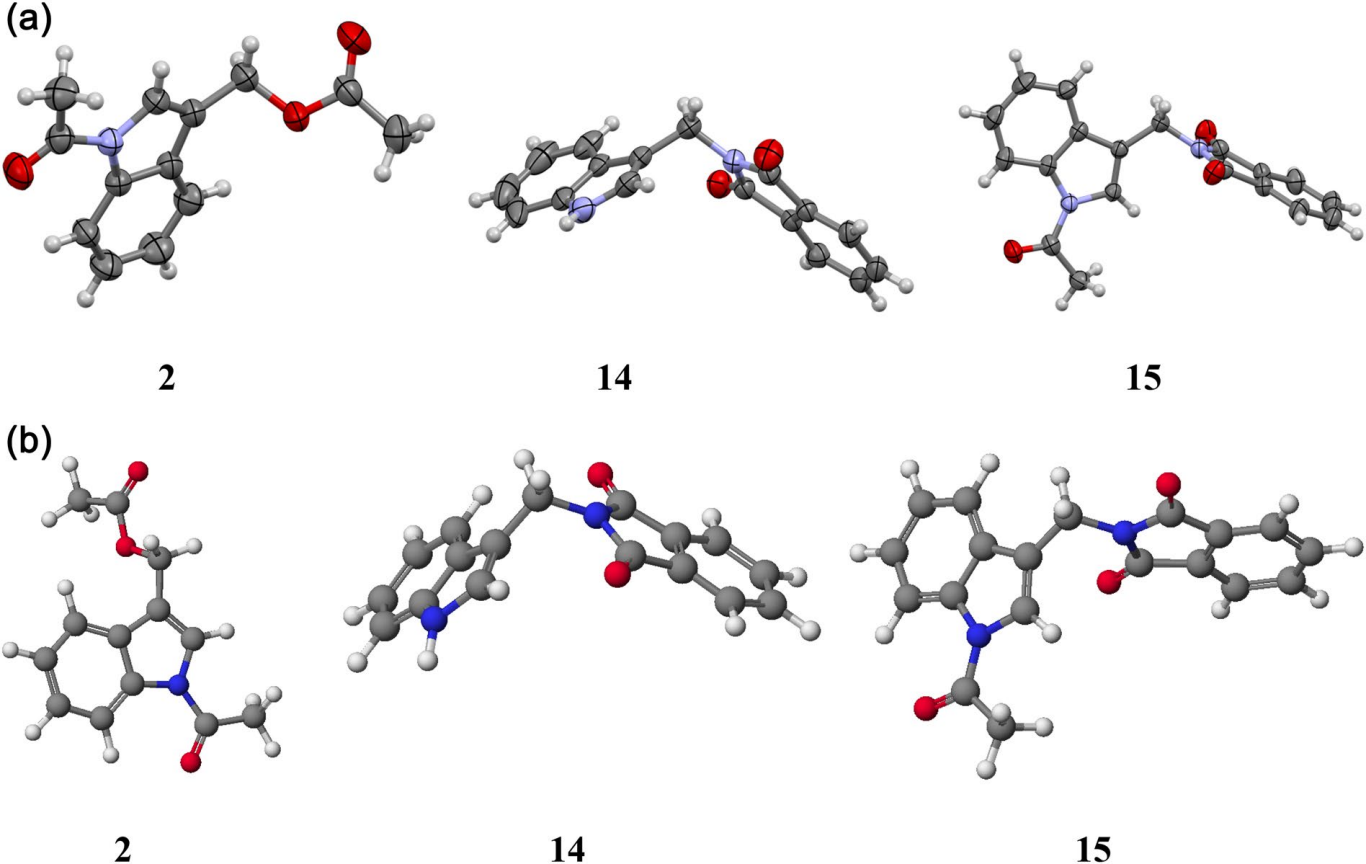
(**a**) Molecules of **2**, **14**, and **15** present in crystals at 293 K. Thermal ellipsoids are drawn at a 40% probability level. H-atoms are presented in arbitrary radii. (Computer graphics program MERCURY)^38^. (**b**) A perspective view of gramine derivatives **2**, **14**, **15** calculated by semiempirical PM5 method (WinMopac 2003)^39^.

Single crystals suitable for X-ray diffraction were obtained for three derivatives (**2**, **14**, **15**). The molecular structures of these compounds are shown in Fig. 2a. The molecules consist of two methylene bridged planar subunits, viz the indole and phthalimide (**14**, **15**) or acetyloxy (**2**) moieties. The molecules are not planar. The indol parts form an angle of 57.0(2), 71.7(1) and 10.3(2)° with the plane containing the methylene C–C and C-O/N bonds (called the methylene plane) while the acetyloxy (in **2**) or phthalimide fragments (in **14** and **15**) form an angle of 18.7(3), 78.1(2) and 78.6(7)° with this methylene plane. Comparison of the closely related structures of **14** and **15** clearly illustrates that introducing the *N-*acetyl substituent to the indole part of the molecule changes the molecular conformation in the solid-state from skewed (**14**) to the one in which the indole ring is close to co-planar. In contrast, the phthalimide ring is nearly perpendicular to the methylene plane, resulting in the phthalimidomethyl group being approximately bisected by the indole plane (**15**). Such conformation seems well suited for attractive C–H(indole)/N–C(methylene) 1,3-dipolar interactions. However, it is expected that this type of molecule does not have a fixed conformation in solution at room temperature (~ 22 °C).

In many works that describe the application of computational methods, one can find information comparing theoretical results with crystallographic structures. Not without significance is the use of computational methods to determine the docking properties^34–36^. We were able to obtain an excellent picture of molecular modeling using semiempirical calculations^37^. All the structures were calculated by the PM5 semiempirical method. Molecular structures of compounds **2**, **14** and **15** (Fig. 2b) have been shown together with crystallographic structures (Fig. 2a) to complete their picture. The calculated structure of the remaining compounds (which do not have crystallographic structures) are shown in supplementary materials (Figure S8).

The calculated parameters for compounds **2**, **14** and **15** are comparable with those determined by the X-ray method, although these two methods describe two different states, i.e., the gas and the solid. Thus, the PM5 semiempirical method is a reliable method of visualization of the structures in the solid-state. This result is in perfect agreement with other findings as well as with our previous calculations.

In silico tests with the Prediction of Activity Spectra (PASS) computer program enables faster material selection for pharmacological tests. The PASS calculations show that the derivatives **5**, **6**, **8**, and **9** are characterized by cytoprotective and membrane-protective properties with predicted activity PA > 63%.

### Antioxidant potential

**Figure 3:**
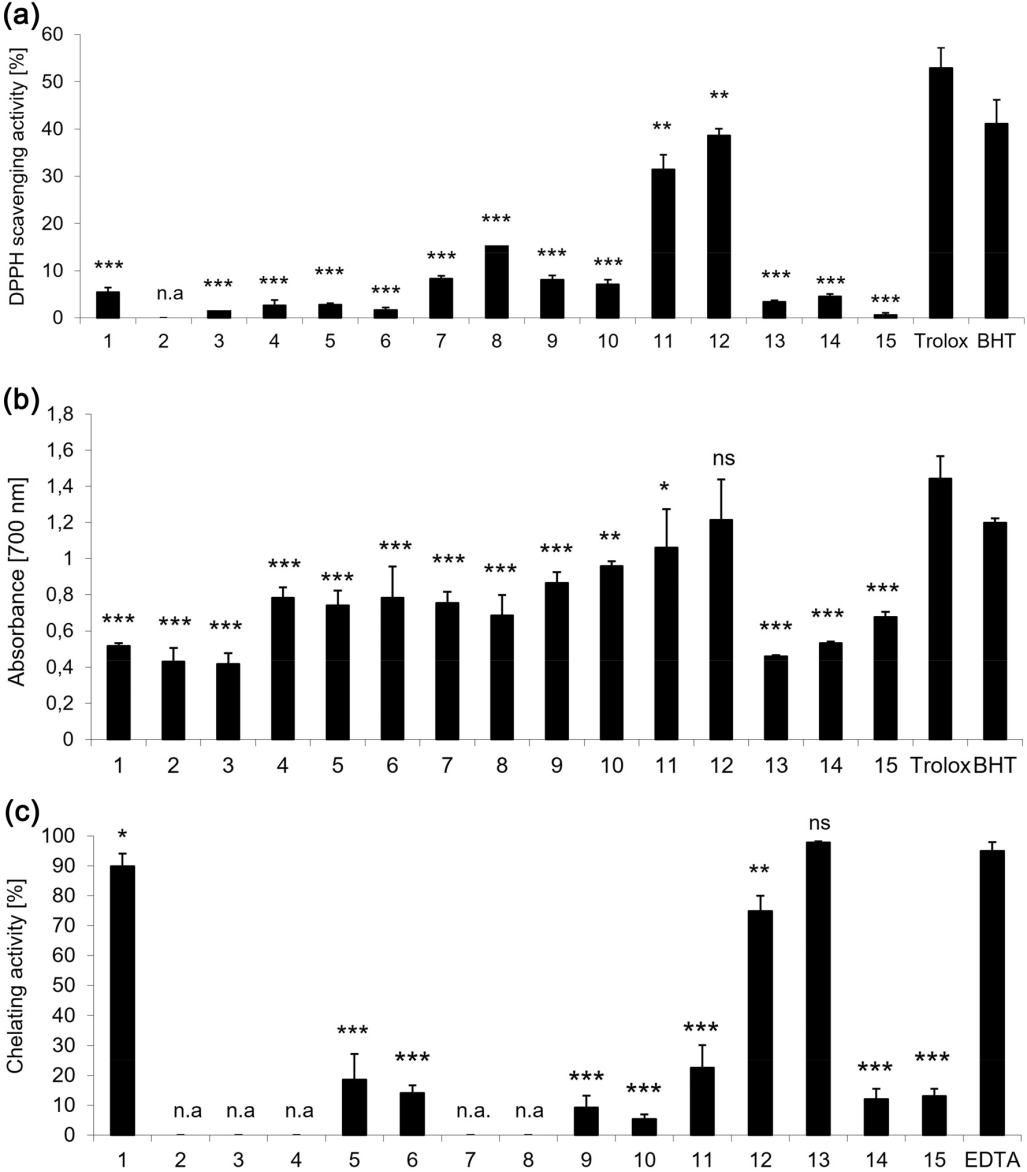
(**a**) DPPH free radical scavenging activity of compounds tested and standard antioxidants Trolox and BHT. (**b**) The Fe^3+^-Fe^2+^ reductive potential of compounds tested, and standard antioxidants Trolox and BHT. (**c**) Ferrous ions chelating activity of derivatives and standard chelating agent EDTA. All compounds were used at a concentration equal to 0.1 mg/mL. The results are presented as the mean value ± standard deviation (***P < 0.001, **P < 0.01, *P < 0.05) in comparison with standard antioxidants Trolox or EDTA, respectively. Inactive derivatives are indicated as n.a. Non statistically significant difference is indicated as ns.

Antioxidants are involved in preventing ROS-dependent cellular damage and, therefore, counteract the development of ROS-related diseases^1^. The antioxidant activity of compounds has been attributed to the various mechanisms, including single electron transfer (SET) and hydrogen atom transfer (HAT) mechanisms. To evaluate the antioxidant activity of indole derivatives and compare their structure-relation mode of actions, DPPH scavenging, Fe^2+^ chelating activity, and reducing ability assays were used. As can be seen in Fig. 3a, the scavenging activity of derivatives **11** (31%) and **12** (38%) was the highest. Compared to the scavenging activity of gramine (**1**) (5%), the antiradical activity of derivatives **11** and **12** was more than 6 and 7 times higher, respectively. Other derivatives showed lower (≤ 16%) or lack of (derivative **2**) free radical-scavenging effectiveness. It has been proved that the presence of indole structure affects the effectiveness of antioxidants. Due to the free electron pair located on it, the heterocyclic nitrogen atom is an active indole redox center^40^. Therefore, the relocation of this electron pair in the aromatic system seems greatly important for indole derivatives antioxidant activity.

As mentioned above, the inactivation of DPPH free radicals by antioxidants can follow two main mechanisms, namely SET and HAT mechanisms, which often occur together^41^.

Our results suggest that the mechanism of DPPH radical scavenging by active gramine derivatives could be explained as the indole rings initial hydrogen or electron abstraction. A single electron is transferred from the nitrogen atom during the SET mechanism, and a cation radical is formed on it. It is also possible to transfer a hydrogen atom (presence of the N–H group) from the antioxidant molecule to the DPPH radical and form a resonance-stabilized indolyl radical. The exact mechanism was proposed by Silceria et al. for C-3 sulfenyl indoles^12^. An essential element of the structure of the analyzed derivatives, which significantly affects the efficiency of scavenging the DPPH radical (except indole ring and N–H group), is the presence of various functional groups with different electronic and lipophilic properties directly connected to the methylene group at the C-3 position.

**Figure 4:**
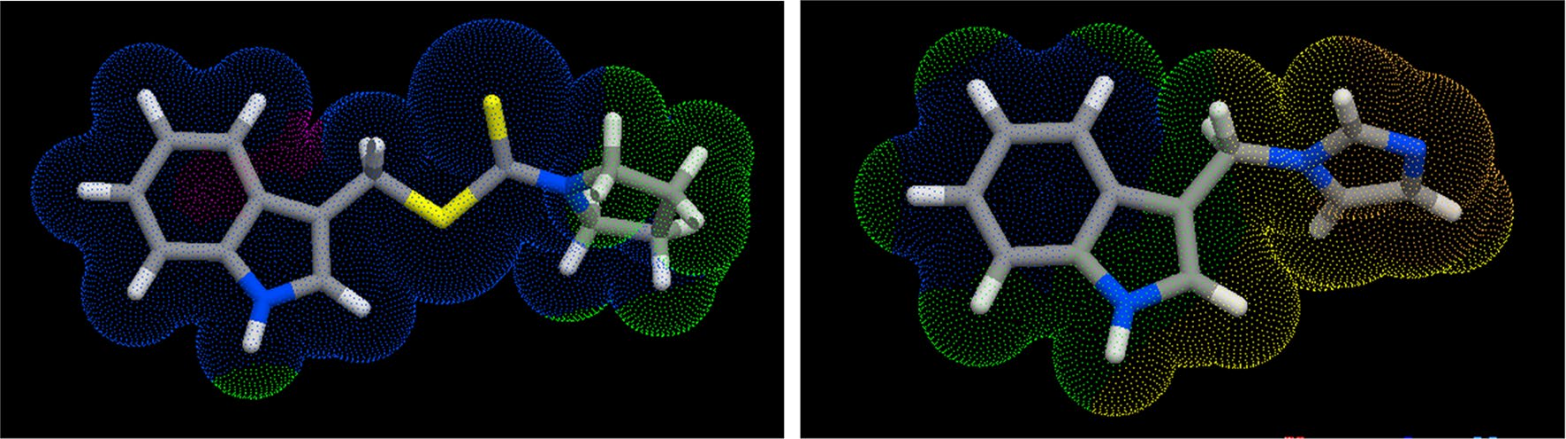
Molecular lipophilicity potentials of derivatives **12** (left) and **13** (right). Different colors indicate the hydrophobic (blue) and hydrophilic (yellow-orange-red) regions (https://www.molinspiration.com).

To visualize the molecular lipophilicity potential (MLP) on the molecular surface, Galaxy Visualizer was used. The MLP of derivatives **12** and **13** were visualized and presented in Fig. 4. These molecules hydrophobic (blue colors) and hydrophilic (yellow-orange-red colors) regions are consistent with the reaction mechanism: –the indole ring is the electron donor part of the molecules, and the ring thus dissipates the radicals. Such a system of electron density in individual molecules makes it possible to explain their mutual structural relations.

The presence of the diindol or pyrrolidinedithiocarbamate moiety makes derivatives **11** and **12** the best radical scavenger. Moreover, these compounds can become resonance-stabilized radicals due to a homolytic cleavage of the C–H bond. In this case, it is symmetrically fuzzy on the molecule. Such hydrogen atom loss from DIM·^+^ (compound **11**) was detailed described by Błoch-Mechkour et al.^42^. On the other hand, in derivatives **13**–**15**, the imidazole and phthalimide rings participate in resonance, which does not stabilize the radical form. The effect of an acetyl group present at the nitrogen atom on a decrease in the antioxidant properties of indole derivatives is well illustrated by comparing the activity of compound **3** with that of indole-3-carbinol, the dopamine-like antioxidant^43^.

Derivatives **11** and **12** also exhibited a significant reducing power at the level statistically comparable (**12**) to the reference antioxidant Trolox (see Fig. 3b). Gramine (**1**) and compounds **2–3** and **13–15** had the lowest reductive potential, whereas the reductive potential for other derivatives **4**–**10** was higher than gramine, regardless of the alkyl chain length or its branching. Thus, they are better than gramine electron donors that react with free radicals and effectively block radical chain reactions. In compounds **4**–**10**, a more stable C-centered radical can be formed by losing a hydrogen atom from the CH_2_ group, as in indole-3-carbinol^42^. H-abstraction from the C-3 side chain was also observed in tryptophan and tryptamine derivatives^40,44^. Introducing an acetyl group at the nitrogen atom in the indole ring prohibits its formation, which results in less active compounds than gramine.

Another important mechanism of antioxidants action is the chelation of transition metals, which promote oxidation by acting as catalysts of free radical reactions. As shown in Fig. 3c, gramine (**1**) itself is an excellent Fe^+2^ chelator (89%). Introduction imidazole ring into its molecule (compound **13**) further increases the chelating activity (97%). Both compounds can complex almost all iron ions in the incubation solution with the same efficiency. The presence of pyrrolidinedithiocarbamate moiety in compound **12** decreases the chelating activity of gramine alone (**1**) by about 15% (from 89 to 74%). Other gramine derivatives did not serve as potent Fe^2+^-chelators. This fact is primarily due to structural reasons, including a substituent on the nitrogen atom (derivatives **2**, **3**, **15**) or the rigid structure of the molecule (derivatives **11** and **14**).

The semiempirical calculations also confirm this relatively low antioxidant activity of most of the gramine derivatives studied. The substitution of the annular nitrogen atom and the angular hydroxyl group in the gramine skeleton causes the heat of formation (HOF) to decrease dramatically. This fact is consistent with our expectations because these reactive groups are blocked in this case. The greater stability of the carbonyl group can explain the lower HOF value for derivative **2** (See Table S2). On the other hand, the free hydroxyl group in derivative **3** and the ring nitrogen atom in derivative **1** increase HOF. Moreover, derivatives **2** and **3** have a blocked nitrogen atom with a relatively large group; hence hydrogen bonds formation are difficult. An interesting observation is the replacement of the acyl group with alkoxy groups (OCH_3_, OC_2_H_5_, OC_3_H_7_, OCH(CH_3_)_2_, OC_4_H_9_, OC_5_H_11_, OC_2_H_4_CH(CH_3_)_2_). The lowest HOF values were observed for derivative **9**, where the OH group is substituted with a straight-chain alkyl. This fact can be explained by van der Waals’s interactions between flat indole rings and properly arranged straight chains. Three of the obtained compounds, namely **11**, **12**, and **13**, have a higher heat of formation than gramine, which may explain their high reactivity. Derivatives **14** and **15** also show low heat of formation, which can be explained as in derivative **2**. The stability of these compounds was confirmed by crystallographic characterization.

### Haemolytic activity and human red blood cells (RBC) shape transformation

The membrane-perturbing activity of all derivatives was evaluated using human RBC in in vitro haemolytic assay. As shown in Table 1, the haemolytic potency of derivatives was the structure- and concentration-dependent. Moreover, the derivatives **7–11** induced the biconcave-discoid human RBC transformation into stomatocytes (cup cells) at the higher concentration used (0.1 mg/mL). None of the derivatives induced haemolysis and distinct RBC shape changes at the lower concentration used (0.025 mg/mL). It can be argued that the presence of branching at the end of the alkyl chain in the molecule determines the haemolytic activity of derivatives at concentration 0.1 mg/mL (haemolytic activity of compounds **7** > **6** and compounds **10** > **9**). Moreover, the dependence of haemolytic activity on the carbon chain length was noticed, namely, the longer the branched carbon chain, the higher haemolytic activity of the compound (haemolytic activity of compounds **10** > **7**).

**Table 1:** The haemolytic activity of compounds studied and the predominated human RBC shape after the standard incubation with compounds (1 h, 37° C).

Compound	Haemolytic Activity (%) at 0.1 mg/mL	Dominate cells shape at 0.1 mg/mL	Haemolytic activity (%) at 0.025 mg/mL	Dominate cells shape at 0.025 mg/mL	Intensity of MC540 at 0.1 mg/mL
Control (0 mg/mL)	2.21 ± 0.57	D	2.14 ± 0.68	D	1.0
**1**	2.92 ± 0.68	D	2.53 ± 0.47	D	1.14
**2**	3.27 ± 0.19	D	1.08 ± 0.81	D	1.08
**3**	8.41 ± 1.51	D	3.78 ± 1.08	D	1.22
**4**	8.16 ± 1.76	D/E	3.02 ± 0.44	D	NA
**5**	12.08 ± 2.41	D	2.85 ± 0.52	D	NA
**6**	5.87 ± 1.25	D	2.91 ± 1.15	DE	1.12
**7**	8.43 ± 1.17	S	3.05 ± 0.96	S/DE	1.54
**8**	8.69 ± 2.35	S	2.47 ± 1.04	DE	1.71
**9**	4.78 ± 1.66	S	2.09 ± 1.22	D/S	1.43
**10**	10.77 ± 3.42	SS	3.06 ± 0.55	D/S	NA
**11**	74.12 ± 8.62	SS/S	3.43 ± 0.51	D/DE	NA
**12**	18.93 ± 4.47	D/S	3.07 ± 0.48	DE	NA
**13**	3.08 ± 0.29	D	2.59 ± 0.26	D	1.16
**14**	3.84 ± 1.33	D	2.14 ± 0.54	D	0.91
**15**	9.82 ± 2.41	DS	2.52 ± 0.73	D	NA

The predominant human RBC shape: D-biconcave-discocytes, control cells, DE-discoechinocytes; E-echinocytes; S-stomatocytes; SS-spherostomatocytes. Haemolytic activity > 5% means the significant membrane-perturbing activity of a compound. NA not analyzed because of haemolysis. Data represent the mean value ± SD from 3 independent experiments (n = 9).

**Figure 5:**
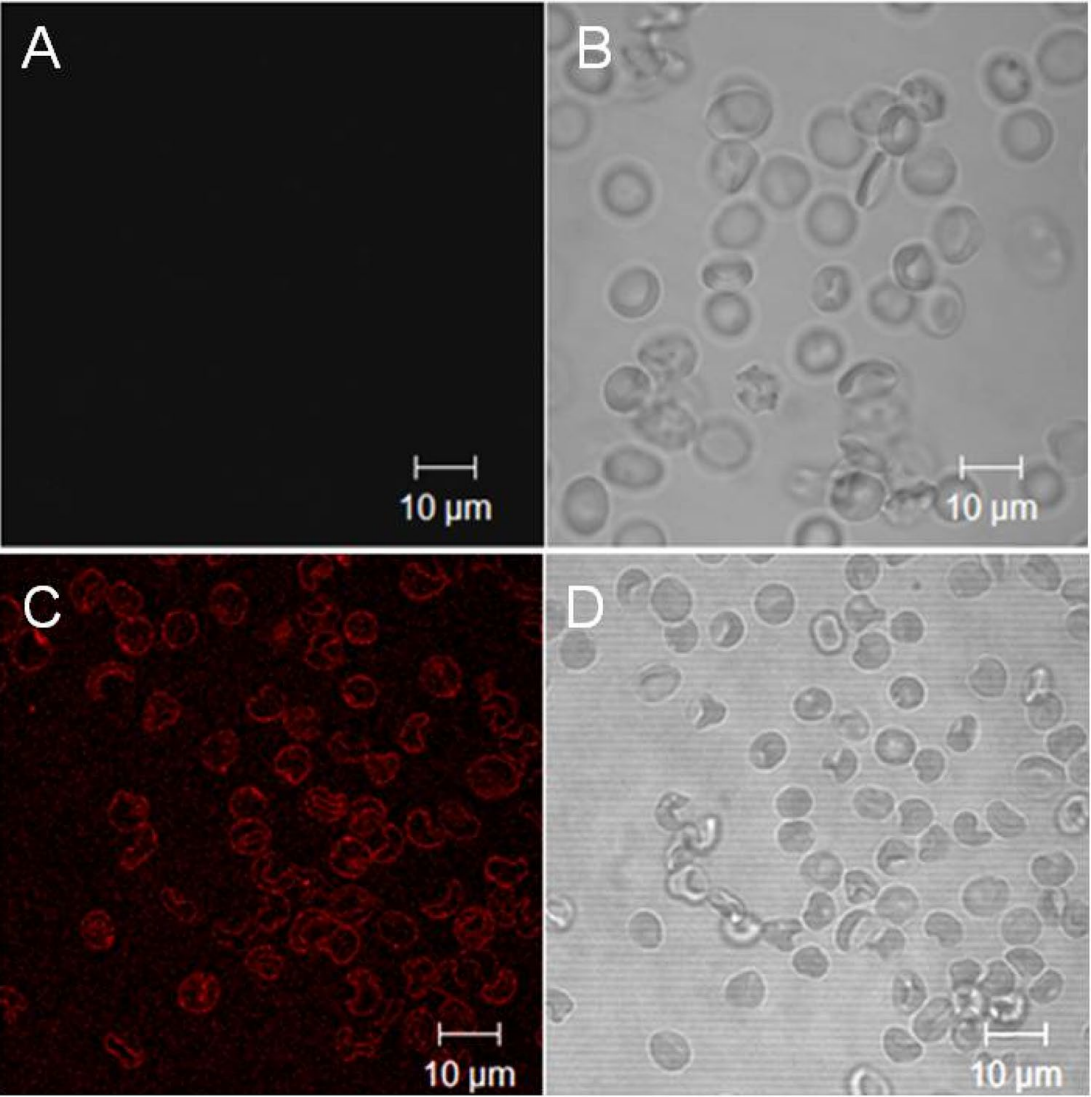
Phospholipids asymmetry and phospholipids packing in RBC membrane analyzed by merocyanine 540 (MC540) binding. Control biconcave-discoid RBC (**A** and **B**) and stomatocytic RBC (**C**, **D**) induced by compound **8** at 0.1 mg/mL. The confocal images (right panel) show fluorescence (in **C**) due to the binding of MC540 to the RBC membrane with disordered lipid packing. The left panel shows the RBC shape in the contrast phase (B-discocytes, D-stomatocytes). Presented RBC (control and compound **8**-treated) were incubated with MC540 at the same conditions. The images show representative data for three independent experiments. Scale bars indicate 10 µm.

To obtain data regarding the influence of gramine derivatives on the RBC membrane molecular structure and phospholipid asymmetry, the confocal microscopy analysis and fluorimetric method after RBC incubation with a heterocyclic chromophore merocyanine 540 (MC540), were applied. MC540 is a sensitive fluorescent probe used to estimate the molecular packing of lipids in the exoplasmic leaflet of RBC membrane^45,46^. The binding of MC540 into the exoplasmic layer of the RBC membrane with disordered lipid packing results in red fluorescence^47^. As shown in Fig. 5A, the control biconcave-discoid RBC (see also Fig. 5B) remained MC540-unstained while derivative **8**-induced stomatocytic RBC (see Fig. 5D) are characterized by the red fluorescence (see Fig. 5C and data summarized in Table 1). However, the differences in fluorescence intensity between the control biconcave-discoid RBC and the compound **8**-incubated RBC are not substantial (compare values in Table 1). At the concentration equal to 0.025 mg/mL, no MC540-stained RBC were observed for all studied compounds (results not shown); therefore, this concentration was selected for further studies. Therefore, it can be stated that gramine and its derivative at a concentration of 0.025 mg/mL do not induce unfavourable changes in the molecular structure of the RBC lipid bilayer.

### Protective activity against induced oxidative haemolysis

**Figure 6:**
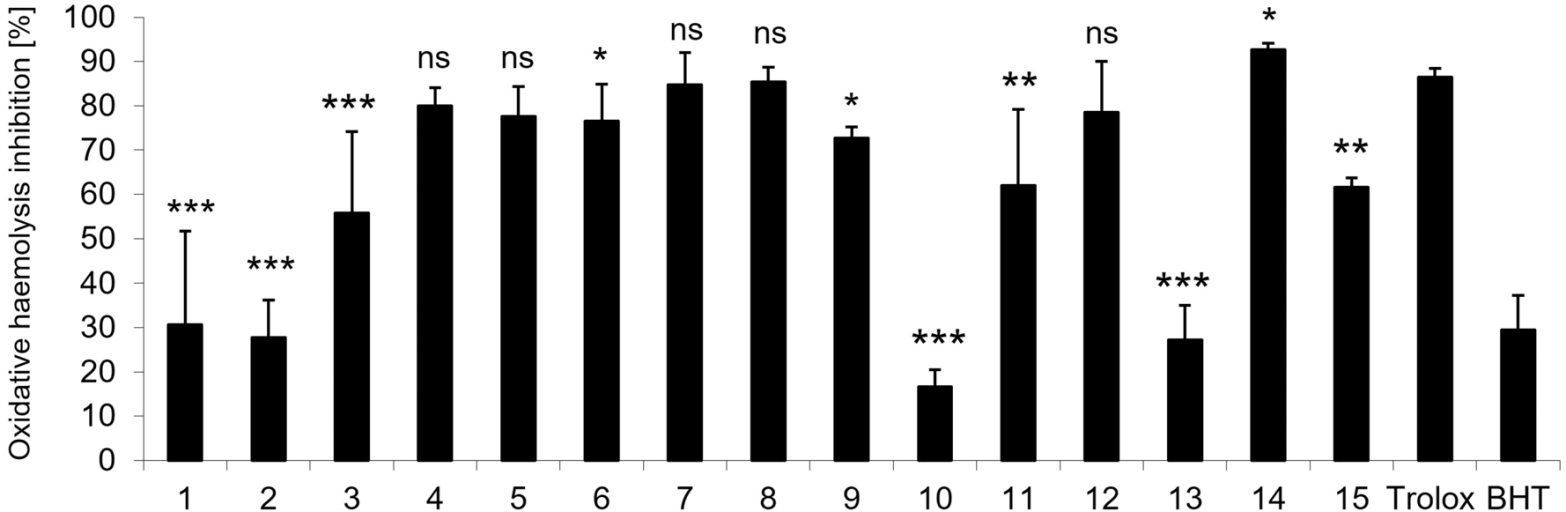
In vitro protective effects of compounds tested and standard antioxidants Trolox and BHT at the concentration of 0.025 mg/mL against 60 mM AAPH-induced haemolysis after 20 min pre-incubation with human RBC. The results (n = 9) are presented as the mean value ± standard deviation (***P < 0.001, **P < 0.01, *P < 0.05) in comparison with standard antioxidant Trolox. Non statistically significant difference is indicated as ns.

The capacity of all new indole-based derivatives to protect human RBC against 2,2'-azobis(2-methylpropionamidine) dihydrochloride (AAPH)-induced oxidative haemolysis was estimated at the concentration of 0.025 mg/mL. As shown in Fig. 6, all derivatives inhibit AAPH-generated oxidative haemolysis in a structure-dependent manner. Gramine (**1**) (30%) and derivatives **2** (27%), and **13** (27%), were as efficient cytoprotective agents as the standard antioxidant BHT (29%). On the other hand, compounds **4**, **5** and **7**, **8** (from 79 to 84%), and **12** (78%) protected RBC against oxidative haemolysis as effectively as the standard antioxidant Trolox (86%). Moreover, the cytoprotective activity of compound **14** (92%) was statistically higher than the activity of Trolox.

Our structure–activity analysis showed that the length of the alkyl chain had no significant impact on the cytoprotective activity of compounds **4**–**8**, all with a weak antioxidant potential estimated in cell-free assays (compare Fig. 3). Interestingly, the cytoprotective activity of compound **12,** with the highest antioxidant potential in cell-free assays, is similar to the cytoprotective activity of compounds **4**, **5** and **7–8**. Moreover, compound **14**, with low antioxidant potential in cell-free assays, shows the highest cytoprotective activity under oxidative stress conditions. The cytoprotective activity of the bioactive compounds without or with low antioxidant potential in cell-free assays can be explained by the beneficial interactions of their molecules with the cell membrane components^11,18^.

**Figure 7:**
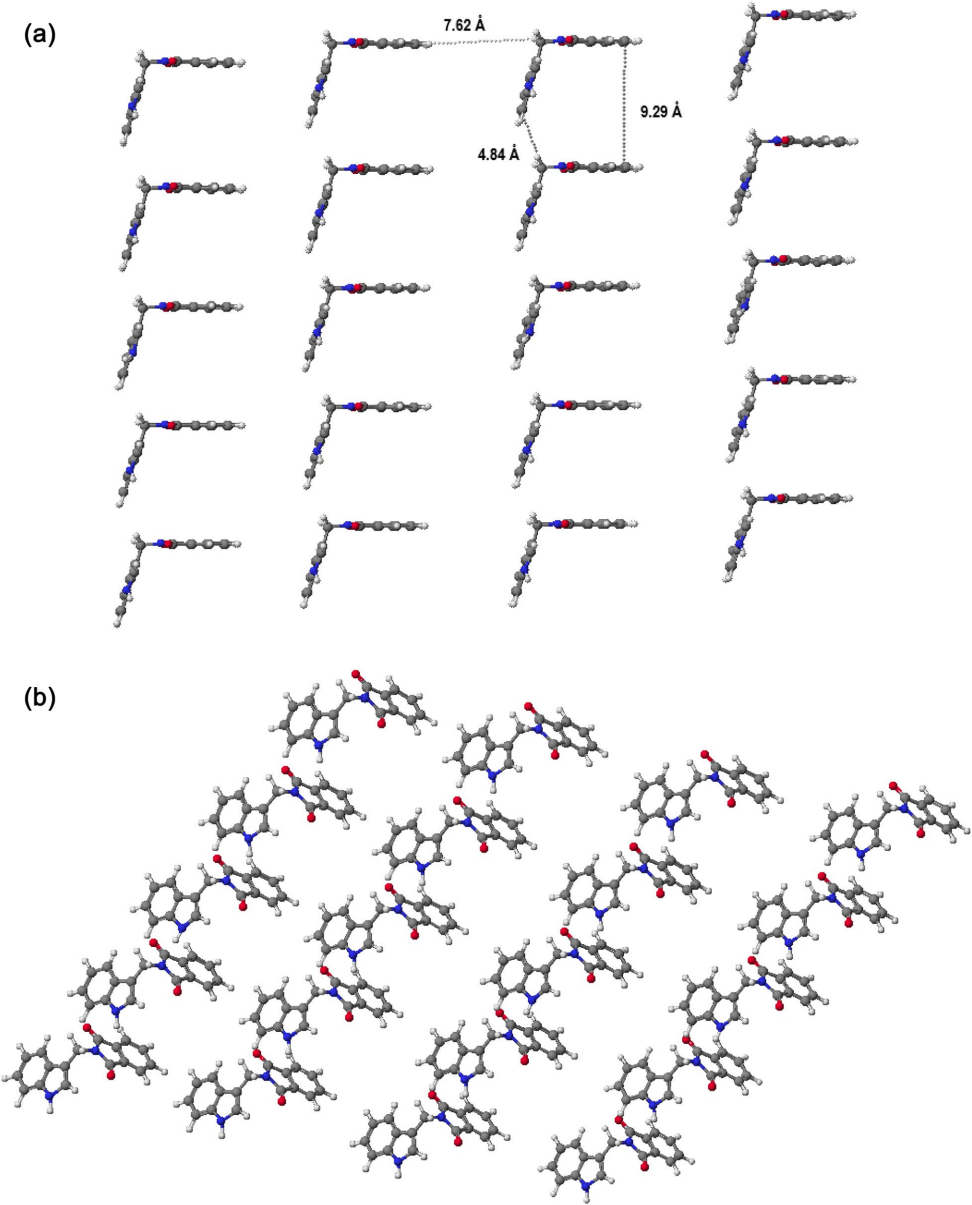
Molecular models of derivative **14** calculated by PM 5 method for twenty molecules in two different perspectives: side view (**a**), top view (**b**) (WinMopac 2003)^39^.

Supramolecular system for compound **14** was calculated to better understand these interactions. The data show the arrangement of molecules in relation to each other (Fig. 7). As a result of the formation of supramolecular systems, micelles are easily formed, which can act as a protective barrier around the RBC membrane against the detrimental effect induced by external factors. In biological systems, they can be formed by the formation of weak interactions such as hydrogen bonds, ion–dipole interactions or van der Waals forces. Figure 7a presents the possibility of creating hydrogen bonds with marked distances between aromatic rings. The heat of formation for such a system is –63.4396 kcal/mol, which means that the molecules can form a specific micelle that protects the cell membrane and the cell in general. Moreover, it has been shown that gramine establishes hydrogen bonds with phosphate groups or the ester groups of the lipid molecules in artificial lipid bilayers^48^. However, electrostatic interactions of gramine with negatively charged phosphate groups are also possible. The overall hydrogen bond number between alkaloids and lipids is only marginally affected by the lipid types. Polar interactions can involve two groups. In derivative **14**, the nitrogen atom in the indole ring remains unblocked, making it a proton donor and can easily form hydrogen bonds with components of the biological membrane. Therefore, the highest cytoprotective activity of derivative **14**, with low antioxidant activity confirmed estimated in the cell-free assays, can be explained by hydrogen bond formation with phosphate groups of lipids in the exoplasmic leaflet of the RBC membrane.

As mentioned before, the interaction of gramine molecules and their derivatives with the lipids of the exoplasmic layer of the RBC membrane does not induce changes in its molecular structure. Furthermore, it results in RBC protection against free radicals adverse effects. This observation is in line with our results showing no effect of all the derivatives on the lipid packing in the RBC membrane. Therefore, the high cytoprotective potential against oxidative haemolysis of derivatives **4–8**, with low antioxidant potential confirmed in the cell-free antioxidant assays, can be explained by their specific interaction with the lipid bilayer of the RBC membrane. Moreover, as have been mentioned before 2.1, PASS analysis indicated the cytoprotective and membrane-protective properties of derivatives **5–6** and **8–9.** These gramine derivatives, including derivative **7**, have an angular oxygen atom in their structure in addition to the annular nitrogen atom, both with electronegativity greater than 3.

## Conclusion

Natural compounds are promising starting molecules to develop new potent drugs or food additives, including antioxidants. Alkaloids constitute one of the largest classes of natural products and one of the most promising being indole alkaloids. Different functional groups at the methylene group at the C-3 position of the indole nucleus have been shown to modulate the antioxidant ability of newly synthesized derivatives. Compound **12** with pyrrolidinedithiocarbamate moiety has significant antioxidant properties confirmed by two antioxidant activity assays. Selected indole derivatives, namely **4–8**, **12**, and **14**, were the most effective cytoprotective agents against oxidative stress-induced haemolysis. The cytoprotective activity of derivatives can be explained by hydrogen bond formation with phosphate groups of lipids in the exoplasmic leaflet of the RBC membrane or electrostatic interactions with negatively charged phosphate groups. Theoretical estimations and experimental data confirm the beneficial interactions between the selected C-3 substituted indole derivatives and the RBC membrane that protect cells against oxidative damage.

## Methods

### Instrumentation and chemicals

The melting points (mp) were obtained with a Büchi SMP-20 apparatus. ^1^H NMR and ^13^C NMR spectra were recorded on a Varian 300/400 spectrometer with CDCl_3_ or DMSO-*d*_*6*_ as the solvent and TMS as the internal standard. Chemical shifts are reported in δ (parts per million) values. EI mass spectra were measured on Bruker 320MS/450GC mass spectrometer. FT-IR spectra were recorded on Nicolet iS 5 (KBr pellets). The elementary analysis of all indole derivatives was carried out on Vario ELIII (Elementar, Germany). TLC analysis was used using Sigma-Aldrich silica gel 60 plates with a fluorescent indicator (254 nm) and visualized with UV or Dragendorff's Reagent. All chemicals or reagents used for syntheses were commercially available. In all reactions, anhydrous solvents were used.

### Synthesis of gramine derivatives

The synthesis of compound **2** is described elsewhere^11^.

#### N-acetyl-indole-3-carbinol (3)

*N,O*-diacetyl-indole-3-carbinol (**2**) (115.5 mg, 0.5 mmol) in water (15 mL) was heated at the reflux temperature for 5 h (TLC: (C_6_H_5_)CH_3_-AcOEt 5:1). The reaction mixture was then poured onto crushed ice and extracted with diethyl ether (3 × 30 mL). The extract was washed with water (3 × 50 mL), brine (100 mL), and dried over KOH (pellets). The solvent was evaporated under reduced pressure to afford the crude product, which was crystallized from H_2_O.

*3-Alkoxy-indole-3-carbinols (****4–10***) were synthesized according to Jump, S. M. et al.^26^.

Compound **2** (231 mg, 1 mmol) was dissolved in 1 mL appropriate alcohol (methanol, *n*-propanol, *iso*-propanol, *n*-butanol, *n*-pentanol, *iso*-pentanol) and 0,5 mL 10% NaOH was added. The mixture was stirred for 24 h (methanol, ethanol), 2 h (*n*-propanol, 2-propanol), 1 h (*n*-butanol) at room temperature (~ 22° C) or reflux (2 h) for *n*-pentanol and *iso*-pentanol (TLC: (C_6_H_5_)CH_3_-AcOEt 5:1). The mixture was cooled in an ice/water bath, added dropwise 35 mL of cold water and put in a refrigerator overnight. The resulting precipitate of **4** and **5** was filtered and crystallized from *n*-hexane. In the case of compounds **6–10**, the cooled reaction mixture was extracted with diethyl ether (3 × 15 mL), washed with water (3 × 50 mL) and brine (100 mL), and then dried over KOH (pellets). After solvent evaporation, the resulting brown oils of **6–10** were obtained.

#### 3,3’-Diindolylmethane DIM (11)

A solution of gramine (174 mg, 1 mmol), ethylenediamine (1 mmol) and 10% NaOH (0.5 mL) was heated at the reflux temperature for 5 h (TLC: MeOH-(CH_3_)_2_CO-NH_4_OH 10:10:1). The reaction mixture was then extracted with diethyl ether (3 × 30 mL). The extract was washed with water (3 × 50 mL), brine (100 mL), and dried over KOH (pellets). The solvent was evaporated under reduced pressure to afford the crude product, which was crystallized from H_2_O.

#### (1H-indol-2-yl)methylpyrrolidine-1-carbodithioate (12)

A solution of gramine (174 mg, 1 mmol) and sodium pyrrolidinedithiocarbamate (507 mg, 3 mmol) in toluene (10 mL) was heated at the reflux for 30 h (TLC: MeOH-(CH_3_)_2_CO-NH_4_OH 10:10:1). The reaction mixture was then poured onto crushed ice and extracted with diethyl ether (3 × 30 mL). The extract was washed with water (3 × 50 mL), brine (100 mL) and dried over KOH (pellets). The solvent was evaporated under reduced pressure to afford the crude product, which was attempts to crystallize from a mixture of *n*-hexane-diethyl ether and petroleum ether-ethyl acetate.

#### 3-Imidazol-1-ylmethyl-indole (13)

A solution of gramine (174 mg, 1 mmol) and imidazole (68 mg, 1 mmol) in toluene (10 mL) was heated at the reflux for 2 h (TLC: MeOH-(CH_3_)_2_CO-NH_4_OH 10:10:1). The evaporation of the solvent and purification of the residue from hexane gave compound **13.**

#### N-Indol-3-ylmethyl-phtalimide (14)

A solution of gramine (174 mg, 1 mmol) and phtalimide (147 mg, 1 mmol) in DMF (10 mL) was heated at the reflux for 18 h (TLC: (C_6_H_5_)CH_3_-AcOEt 5:1). The reaction mixture was then poured onto crushed ice and extracted with diethyl ether (3 × 30 mL). The extract was washed with water (3 × 50 mL), brine (100 mL), and dried over KOH (pellets). The solvent was evaporated under reduced pressure to afford the crude product, which was crystallized from ethanol.

#### N-(N’-acetylindol-3-yl)methyl-phtalimide (15)

A mixture of compound **14** (100 mg, 0.36 mmol) and potassium carbonate (25 mg, 0.18 mmol) was dissolved in acetic anhydride (1.8 mmol, 0.17 mL) and heated at the reflux for 2 h (TLC: (C_6_H_5_)CH_3_-AcOEt 5:1). The reaction mixture was then poured onto crushed ice and extracted with chloroform (3 × 30 mL). The extract was washed with water (3 × 50 mL), brine (100 mL), and dried over anh. NaSO_4_. Following evaporation, the mixture was purified chromatographically on Florisil (compound was eluted successively with diethyl ether (50 ml) and chloroform (50 mL) to give compound **15**, which was crystallized from acetone.

### X-ray data collection and refinement of the structures 2, 14, and 15

X-ray diffraction data were collected at 295 K for **2, 14** and 1**5** on SuperNova kappa-geometry diffractometer equipped with copper X-ray source using CrysAlisPro software CrysAlisPRO, Agilent Technologies, Yarnton^49^. Absorption was corrected for by multi-scan methods, (empirical absorption correction using spherical harmonics, implemented in SCALE3 ABSPACK scaling algorithm). The structures were solved by direct methods using SHELXS-97^50^, and refined by full-matrix least-squares calculations on *F*^2^ with SHELXL-2014/7^51^. Anisotropic displacement parameters were refined for all non-hydrogen atoms. H atoms were positioned geometrically and allowed to ride on their respective parent atoms, with Uiso(H) = 1.2 Ueq(C) and C-H = 0.93 Å for aryl, C-H = 0.97 Å for methylene and C-H = 0.96 Å for methyl, except for the hydrogen atom bonded to N1 in **14** which was located on a difference Fourier map and allowed to refine freely. The crystal structure of **14** is noncentrosymmetric, and the value of the Flack parameter *x* = 0.08(5) indicated^52^ the proper assignment of the absolute structure of the crystal. MERCURY^38^, a computer graphics program, was used to prepare drawings. Crystal data and data collection and refinement details are collected in Table S1 (supplementary part).

### Calculation

PM5 semiempirical calculations were performed using the WinMopac 2003 program^39,53,54^.

### In silico study

Potential pharmacological activities of the synthesized compounds have been determined based on computer-aided drug discovery approach with in silico Prediction of Activity Spectra for Substances (PASSs) program (Pharma Expert Predictive Services©2011–2013, Version 2.0)^55–57^.

### Antioxidant assays

#### DPPH^•^ free radical scavenging activity

1,1-diphenyl-2-picryl-hydrazyl free radical (DPPH^•^) shows absorbance at 517 nm, which decreases upon reduction by an antioxidant. In brief, 0.1 mmol solution of DPPH^·^ was prepared in ethyl alcohol, and 0.2 mL of this solution was added to 0.2 mL of the compound tested at 0.1 mg/mL concentration in ethyl alcohol and vortexed. Trolox (6-hydroxy-2,5,7,8-tetramethylchroman-2-carboxylic acid, a water-soluble analog of vitamin E) and BHT (butylated hydroxytoluene) were used as the reference antioxidants. The samples were incubated in the dark for 30 min at room temperature (~ 22 °C, RT). Following incubation, the absorbance (Abs) was measured at 517 nm in a spectrophotometer. The percent DPPH^•^ scavenging effect was calculated using the equation:

$${\text{DPPH}}^{ \cdot } \;{\text{scavenging}}\;{\text{activity}}\left( \% \right) = \left[ {({\text{Abs}}_{0} {-}{\text{Abs}}_{l} )/{\text{Abs}}_{0} } \right] { \times }100$$

where Abs_0_ is the absorbance of the control reaction without compounds tested and Abs_1_ is the absorbance in the presence of compounds tested. Each sample was made in triplicate, and three independent experiments were performed.

#### Fe^3+^ reducing power assay

Reducing power was determined by the direct reduction of Fe^3+^(CN^−^)_6_ to Fe^2+^(CN^−^)_6_ and determined by measuring absorbance resulted from the formation of the Perl’s Prussian Blue complex following the addition of excess ferric ions (Fe^3+^). 0.1 mg/mL concentration of compound tested in 0.06 mL of distilled water were gently mixed with 0.1 mL of 0.20 MPBS (pH 6.6) and 0.1 mL of 1% potassium ferricyanide [K_3_Fe(CN)_6_]. Trolox and BHT were used as the reference compounds. The samples were vortexed and incubated for 20 min at 50 °C. Following incubation, 0.1 mL of 10% trichloroacetic acid was added to the samples to acidify the reaction medium. Finally, 0.040 mL 0.6 M FeCl_3_ was added to the medium, and the absorbance (Abs) was measured at 700 nm in a spectrophotometer. The increase in absorbance value of the reaction medium corresponded to a more effective reduction capability of the compound tested. Each sample was made in triplicate, and three independent experiments were performed.

#### Ferrous ions (Fe^2+^) chelating activity

Ferrous ions (Fe^2+^) chelating activity was evaluated by inhibition of the formation of Fe^2+^-ferrozine complex after incubation of the compounds tested with Fe^2+^. The Fe^2+^-chelating ability of compounds tested was determined by the absorbance of the ferrous ion-ferrozine complex at 562 nm. In brief, 0.1 mg/mL concentration of the compounds tested in 0.2 mL ethyl alcohol were added to a solution of 0.6 mM FeCl_2_ (0.05 mL). EDTA (ethylenediaminetetraacetic acid) was used as the standard EDTA chelating agent. The reaction was started by the addition of 5 mM ferrozine (0.05 mL) in ethyl alcohol and shaken vigorously immediately. The samples were stored for 10 min at RT. Following incubation, the absorbance (Abs) of the solutions was measured at 562 nm in a spectrophotometer. The percentage of inhibition of ferrozine–Fe^2+^ complex formation was calculated using the equation:

$${\text{Fe}}^{2 + } \;{\text{chelating}}\;{\text{effect}}\left( \% \right) = \left[ {1 - \left( {{\text{Abs}}_{1} /{\text{Abs}}_{0} } \right)} \right] { \times }100$$

where Abs_0_ is the absorbance of the sample without the tested compound and Abs_1_ is the absorbance in the presence of the compound tested. Each sample was made in triplicate, and three independent experiments were performed.

### Human erythrocyte preparation

All methods were carried out following relevant guidelines and regulations, and the Bioethics Committee approved all experimental protocols for Scientific Research at the Medical University of Poznań (agreement no. ZP/907/1002/18). Human red blood cells concentrates were purchased from Blood Bank in Poznań without any contact with blood donors.

The erythrocytes were washed three times (3000 rpm, 10 min, + 4 °C) in 7.4 pH phosphate buffered saline (PBS: 137 mM NaCl, 2.7 mM KCl, 10 mM Na_2_HPO_4_, 1.76 mM KH_2_PO_4_) supplemented with 10 mM glucose. After washing, RBC were suspended in the PBS buffer at 1.65 × 10^9^ cells/mL, stored at + 4 °C, and used within 5 h.

### Haemolysis assay under the compounds tested

The cytotoxic activity of the compounds tested was determined by a standard haemolytic assay according to Mrówczyńska and Hägerstrand^58^. Briefly, RBC (1.65 × 10^8^ cells/mL, ~ 1.5% haematocrit) were incubated in PBS buffer (7.4 pH) supplemented with 10 mM glucose and containing compounds tested in different concentrations (0.025 or 0.1 mg/mL) for 60 min at 37 °C in a shaking water bath. Samples with RBC incubated in PBS without compounds tested were taken as the control. Each sample was repeated three times, and the experiments were repeated 3 times with RBC from different donors. After incubation, the RBC suspensions were centrifuged (3000 rpm, 10 min), and the degree of haemolysis was estimated by measuring the absorbance (Ab) of the supernatant at 540 nm. The results were expressed as a percentage (%) of haemolysis which was determined using the following equation:

$${\text{haemolysis}}\% = \left( {{\text{sample}}\;{\text{Ab}}/{\text{positive}}\;{\text{control}}\;{\text{Ab}}} \right) { \times }100$$

were positive control is Ab of supernatant of RBC in ice-cold H_2_O.

### Microscope studies of erythrocytes shape transformation

Following incubation with compounds at sublytic concentration (0.025 mg/mL), cells were fixed in 5% paraformaldehyde (PFA) plus 0.01% glutaraldehyde (GA) for 1 h at RT. Fixed cells were washed by exchanging of supernatant with PBS. After washing, erythrocytes were settled on polylysine-treated (0.1 mg/mL, 10 min, RT) cover glasses and mounted on 80% glycerol. The cover slips were sealed with nail polish. A large number of cells in several separate experimental samples were studied for MC540 binding using a Zeiss LSM 510 (AXIOVERT ZOOM) confocal microscope (100 × /1.4 aperture immersion oil objective, 10 × ocular). Images were acquired using the Zeiss LSM Image Browser program. RBC shape was estimated according to the Bessis classification^59^.

### Human erythrocytes lipid membrane asymmetry and fluidity

The effect of compounds on the erythrocyte membrane lipid asymmetry and its fluidity was evaluated from the binding of the fluorophore merocyanine (MC540) with the cell membrane lipid bilayer according to^35^ with the same modifications. Briefly, following incubation at sublytic concentration of compounds (0.025 mg/ml) for 60 min at 37° C, erythrocytes were stained with 17.5 µM MC540 in the presence of 0.15% BSA for 5 min in the dark at RT. After centrifugation (4000 rpm, 5 min, + 4° C), the supernatant was removed, and RBC were resuspended in PBS and fixed with 5% paraformaldehyde plus 0.01% glutaraldehyde (30 min, RT, in the dark). After washing in PBS, RBC were settled on polylysine-treated (0.1 mg/mL, 10 min, RT) cover glasses and mounted on 80% glycerol. The cover slips were sealed with nail polish. A large number of cells in several separate experimental samples were studied for MC540 binding using a Zeiss LSM 510 (AXIOVERT ZOOM) confocal microscope (100 x/1.4 aperture immersion oil objective, 10 × ocular) with an excitation wavelength of 540 nm and an emission wavelength of 580 nm. Images were acquired using the Zeiss LSM Image Browser program. To estimate the relative fluorescence of RBC membrane-bound MC540, cells were treated as above. Following washing (4000 rpm, 5 min, + 4° C), cells were resuspended in PBS (haematocrit ~ 0.02%) and immediately assayed for the fluorescence (λex = 540 nm, λem = 580 nm). The fluorescence of the supernatant of every sample was subtracted from the fluorescence of the RBC suspension and fluorescence intensity of each sample was calculated assuming that the value of the control sample was equal to the value 1.

### Inhibition of the free-radical-induced haemolysis

RBC (1.65 × 10^8^ cells/mL, ~ 1.5% haematocrit) were incubated in PBS buffer (pH 7.4) supplemented with 10 mM glucose and containing compounds tested in the sublytic concentration (0.025 mg/mL) for 20 min at 37 °C in a shaking water bath. After pre-incubation, 2,2'-azobis(2-methylpropionamidine) dihydrochloride (AAPH) was added at the final concentration of 60 mM. Samples were incubated for the next 4 h at 37 °C in a shaking water bath. Erythrocytes incubated in PBS only and in the presence of AAPH were taken as the controls. After incubation, the erythrocyte suspensions were centrifuged (4000 rpm, 5 min, + 4° C), and the degree of haemolysis was determined by measuring the absorbance (Ab) of the supernatant at 540 nm in a spectrophotometer. The percentage of inhibition was calculated using the following equation:

$${\text{Inhibition}}\;{\text{of}}\;{\text{haemolysis}}\left( \% \right) = 100{-}\left[ {\left( {{\text{Ab}}_{{{\text{sample}}}} {-}{\text{Ab}}_{{{\text{blank}}}} /{\text{Ab}}_{{{\text{control}}}} {-}{\text{Ab}}_{{{\text{blank}}}} } \right) { \times }100} \right]$$

where Ab_sample_ is the absorbance value of supernatant obtained from samples incubated with compounds tested, Ab_blank_ is the absorbance of supernatant obtained from samples without compounds tested and AAPH, and Ab_control_ is the absorbance of supernatant obtained from samples with AAPH and in the absence of compound tested. Each sample was made in triplicate and the results are presented as a mean value ± SD value of three independent experiments with RBC from different donors (n = 9).

### Statistical analysis

For antioxidant and cytoprotective properties, data were plotted as the mean value ± standard deviation (SD) of the results of three independent experiments, with every sample in triplicate (n = 9). A paired *t*-Student test was used to compare the derivatives activity with the activity of the standard antioxidants Trolox or EDTA, respectively. Trolox was selected for statistical analysis due to its higher antioxidant activity than BHT. Statistical significance was defined as *P* < 0.05. Inactive compounds were indicated as n.a. Non statistically significant difference was indicated as ns.

## Supplementary Information

Supplementary Information.

## Data Availability

Crystallographic dates CCDC 1485335-1485337 contains supplementary crystallographic data for the structure **2**, **14** and **15**. These data can be obtained free of charge at www.ccdc.cam.ac.uk/conts/retrieving.html [or from the Cambridge Crystallographic Data Centre (CCDC), 12 Union Road, Cambridge CB2 1EZ, UK; fax: +44(0) 1223 336 033; email: deposit@ccdc.cam.ac.uk].

## References

[1] Juan CA, de la Pérez LJM, Plou FJ, Pérez-Lebeña E (2021). The chemistry of reactive oxygen species (ROS) revisited: outlining their role in biological macromolecules (DNA, lipids and proteins) and induced pathologies. Int. J. Mol. Sci..

[2] Suzen S, Cihaner SS, Coban T (2012). Synthesis and comparison of antioxidant, properties of indole-based melatonin analogue indole amino acid derivatives. Chem. Biol. Drug Des..

[3] Süzen, S. Antioxidant activities of synthetic indole derivatives and possible activity Mechanisms in *Bioactive Heterocycles V. Topics in Heterocyclic Chemistry* (ed. Khan, M. T. H.) 145–178 (Springer, Berlin, Heidelberg, 2007).

[4] Liu F, Ng TB (2000). Antioxidative and free radical scavenging activities of pineal indoles. J. Neural Transm..

[5] Suzen S, Ateş-Alagoz Z, Demircigil T, Ozkan SA (2001). Synthesis and analytical evaluation by voltammetric studies of some new indole-3-propionamide derivatives, II. Farmaco.

[6] Kruk I (2007). In vitro scavenging activity for reactive oxygen species by N-substituted indole-2-carboxylic acid esters. Luminescence.

[7] Mehta, J., Rayalam, S. & Wang, X. Cytoprotective effects of natural compounds against oxidative stress. *Antioxidants* **7**, 147 (2018) 10.3390/antiox7100147 10.3390/antiox7100147 PMC6210295 30347819

[8] Mohamed BG, Abdel-Alim MA-A, Hussein MA (2006). Synthesis of 1-acyl-2-alkylthio-1,2,4-triazolobenzimidazoles with antifungal, anti-Inflammatory and analgesic effects. Acta Pharm..

[9] Xiu-Juan Y (2017). Bioactivity-guided synthesis of gramine derivativesas new MT1 and 5-HT1A receptors agonists. J. Asian Nat. Prod. Res..

[10] Kozanecka-Okupnik W (2018). Spectroscopy, molecular modeling and anti-oxidant activity studies on novel conjugates containing indole and uracil moiety. J. Mol. Struct..

[11] Kozanecka-Okupnik W (2020). New triazole-bearing gramine derivatives – synthesis, structural analysis and protective effect against oxidative haemolysis. Nat. Prod. Res..

[12] Silveira CC (2013). Synthesis and antioxidant activity of new C-3 sulfenyl indoles. Tetrahedron Lett..

[13] Bian X, Wang Q, Ke C, Zhao G, Li Y (2013). A new series of N2-substituted-5-(*p*-toluenesulfonylamino)phthalimide analogues as α-glucosidase inhibitors. Bioorg. Med. Chem. Lett..

[14] Porretta GC (1985). Chemotherapeutic agents with an imidazole moiety. I. Synthesis and antifungal activities of 1-aryl-4-p-nitrophenylimidazoles. II Farmaco Edizione Scientifica..

[15] Satyanarayana VSV, Sivakumar A, Ghosh AS (2011). Synthesis, characterization of some new five membered heterocycles based on imidazole moiety and their applications on therapeutics. Lett. Drug Des. Discov..

[16] Qiu M (2013). Pyrrolidine dithiocarbamate inhibits herpes simplex virus 1 and 2 replication, and its activity may be mediated through dysregulation of the ubiquitin-proteasome system. J. Virol..

[17] Zheng X (2014). Synergistic effect of pyrrolidine dithiocarbamate and cisplatin in human carcinoma. Reprod. Sci..

[18] Jasiewicz B (2016). Antioxidant properties of thio-caffeine derivatives: Identification of the newly synthesized 8-[(pyrrolidin-1-ylcarbonothioyl)sulfanyl]caffeine as antioxidant and highly potent cytoprotective agent. Bioorg. Med. Chem. Lett..

[19] Greco I (2020). Correlation between hemolytic activity, cytotoxicity and systemic in vivo toxicity of synthetic antimicrobial peptides. Sci. Rep..

[20] Jasiewicz B (2018). Antioxidant and cytotoxic activity of new di- and polyamine caffeine analogues. Free Rad. Res..

[21] Malczewska-Jaskóła K, Jasiewicz B, Mrówczyńska L (2016). Nicotine alkaloids as antioxidant and potential protective agents against in vitro oxidative haemolysis. Chem. Biol. Interact..

[22] Chen J (2020). Structure-antioxidant activity relationship of methoxy, phenolic hydroxyl, and carboxylic acid groups of phenolic acids. Sci. Rep..

[23] Quick, J., Saha, B. & Driedger, P. E. Protein kinase C modulators. Indolactams. 1. Efficient and flexible routes for the preparation of (−)−indolactam V for use in the synthesis of analogs. *Tetrahedron Lett*., **35**, 8549–8552 (1994).

[24] Diker K, Maindreville D, Lévy J (1999). Synthesis and resolution of a *C*_*2*_-symmetrical indolo-2,3-quinodimethane dimer. Tetrahedron Lett..

[25] Jones DT, Artman GD, Williams RM (2007). Coupling of activated esters to gramines in the presence of ethyl propiolate under mild conditions. Tetrahedron Lett..

[26] Jump SM (2008). N-Alkoxy derivatization of indole-3-carbinol increases the efficacy of the G1 cell cycle arrest and of I3C-specific regulation of cell cycle gene transcription and activity in human breast cancer cells. Biochem. Pharmacol..

[27] Chen X, Fan H, Zhang S, Yu C, Wang W (2016). Facile installation of 2-reverse prenyl group into indoles by a tandem N-alkylation aza-cope rearrangement reaction and its application in synthesis. Chem. Eur. J..

[28] Le Borgne M (1997). Synthesis and *In Vitro* Evaluation of 3-(1-Azolylmethy1)- 1*H*-indoles and 3(1-Azolyl-l-phenylmethyl)-1*H*-indoleass Inhibitors of P450 arom. Arch. Pharm. Pharm. Med. Chem..

[29] Csomós P, Fodor L, Sohár P, Bernáth G (2005). Synthesis of thiazino[6,5-b]indole derivatives, analogues of the phytoalexin cyclobrassinin. A new method for preparation of 3-aminomethylindole. Tetrahedron.

[30] Iwao M, Motoi O (1995). Methodology for the efficient synthesis of 3,4-differentially substituted indoles. Fluoride ion-induced elimination-addition reaction of 1-triisopropylsilylgramine methiodides. Tetrahedron Lett..

[31] Takechi H, Machida M, Kanaoka Y (1988). Intramolecular photoreactions of phthalimide-alkene systems. O Formation of N-(ω-indol-3-ylalkyl)phthalimides. Chem. Pharm. Bull..

[32] Abe T (2013). One-pot construction of 3,30-bisindolylmethanes through bartoli indole synthesis. Org. Lett.

[33] Pillaiyar, T., Gorska,E., Schnakenburg, G., Müller, Ch. E. General Synthesis of Unsymmetrical 3,3′-(Aza)diindolylmethane Derivatives. *J. Org. Chem*. **83**, 9902−9913 (2018). 10.1021/acs.joc.8b01349 30025207

[34] Milanović, Ž. B. et al. Synthesis and comprehensive spectroscopic (X-ray, NMR, FTIR, UV-Vis), quantum chemical and molecular docking investigation of 3-acetyl-4-hydroxy-2-oxo-2H-chromen-7-yl acetate, *J. Mol. Struct*. **1225**, 129256 (2021).

[35] Milenković D (2020). Vibrational and Hirshfeld surface analyses, quantum chemical calculations, and molecular docking studies of coumarin derivative 3-(1-m-toluidinoethylidene)-chromane-2,4-dione and its corresponding palladium(II) complex. J. Mol. Struct..

[36] Dimić, D. et al. Synthesis and characterization of 3-(1-((3,4-dihydroxyphenethyl)amino)ethylidene)-chroman-2,4-dione as a potential antitumor agent. *Oxid. Med. Cell Longev*. **2019**, 2069250. 10.1155/2019/2069250 PMC6393868 30906500

[37] Pospieszny T, Pakiet M, Kowalczyk I, Brycki B (2016). Design, synthesis and application of new bile acid ligands with 1,2,3-triazole ring. Supramol. Chem..

[38] Bruno, I. J. et al. *Acta Crystallogr*.* Sect. B* **58***,* 389–397 (2002). 10.1107/s0108768102003324 12037360

[39] CAChe 5.04 User Guide, Fujitsu Chiba, Japan (2003).

[40] Estevão MS (2010). Antioxidant activity of unexplored indole derivatives: synthesis and screening. Eur. J. Med. Chem..

[41] Brand-Williams W, Cuvelier ME, Berset C (1995). Use of a free radical method to evaluate antioxidant activity. LWT Food Sci. Technol..

[42] Błoch-Mechkour (2010). A. Radicals and radical ions derived from indole, indole-3-carbinol and diindolylmethane. J. Phys. Chem. A.

[43] Quan VV, Mai VB, Pham CN, Mechler A (2019). Hydroxyl radical scavenging of indole-3-carbinol: a mechanistic and kinetic study. ACS Omega.

[44] Estevão MS, Carvalho LC, Ferreira LM, Fernandes E, Manuel M, Marques B (2011). Analysis of the antioxidant activity of an indole library: cyclic voltammetry versus ROS scavenging activity. Tetrahedron Lett..

[45] Kozanecka W, Mrówczyńska L, Pospieszny T, Jasiewicz B, Gierszewski M (2015). Synthesis, spectroscopy, theoretical and biological studies of new gramine-steroids salts and conjugates. Steroids.

[46] Włoch A (2016). Physical effects of buckwheat extract on biological membrane in vitro and its protective properties. J. Membrane Biol..

[47] Pruchnik H (2018). An In Vitro Study of the Effect of Cytotoxic Triorganotin Dimethylaminophenylazobenzoate Complexes on Red Blood Cells. J. Membrane Biol..

[48] Lebecque, S. *et al*. Interaction between the barley allelochemical compounds gramine and hordenine and artificial lipid bilayers mimicking the plant plasma membrane. *Sci. Rep*. **8**, 9784; 10.1038/s41598-018-28040-6 (2018). 10.1038/s41598-018-28040-6 PMC6023908 29955111

[49] CrysAlisPRO, Agilent Technologies, Yarnton, Oxfordshire, England, 2014.

[50] Sheldrick GM (2008). A short history of SHELX. Acta Crystallogr. A.

[51] Sheldrick GM (2015). Crystal structure refinement with SHELXL. Acta Crystallogr. C Struct. Chem..

[52] Flack HD, Bernardinelli G (2000). Reporting and evaluating absolute-structure and absolute-configuration determinations. J. Appl. Cryst..

[53] Stewart, J. J. P. Optimization of parameters for semiempirical methods. II Extension of PM3 to Be, Mg, Zn, Ga, Ge, As, Se, Cd, In, Sn, Sb, Te, Hg, Tl, Pb, and Bi. *J. Comp. Chem.* **12**, 320–341 (1991).

[54] Stewart JJP (1989). Optimization of parameters for semiempirical methods I, Method. J. Comput. Chem..

[55] Pharma Expert Predictive Services online Way2Drug.com©2011–2020, Version 2.0, Available online: http://www.pharmaexpert.ru/PASSOnline/

[56] Poroikov VV, Filimonov DA, Borodina YV, Lagunin AA, Kos A (2000). Robustness of biological activity spectra predicting by computer program PASS for non-congeneric sets of chemical compounds. J. Chem. Inf. Comput. Sci..

[57] Poroikov VV, Filimonov DA (2002). How to acquire new biological activities in old compounds by computer prediction. J. Comput. Aided. Mol. Des..

[58] Mrówczyńska L, Hägerstrand H (2009). Platelet-activating factor interaction with the human erythrocyte membrane. J. Biochem. Mol. Toxicol..

[59] Bessis, M. Red Cell Shape (eds.: Bessis, M., Weed, R. I., Leblond, P. F.) (Springer, Berlin, 1973).

