# Supplementary material for: Synthesis, antioxidant and cytoprotective activity evaluation of C-3 substituted indole derivatives
Source: Sci Rep. 2021 Jul 29;11:15425. doi: 10.1038/s41598-021-94904-z (PMC8322387; doi:10.1038/s41598-021-94904-z)
Supplement: Supplementary file 1 — Supplementary Information. [file 41598_2021_94904_MOESM1_ESM.pdf]

Supplementary data

Synthesis, antioxidant and cytoprotective activity evaluation of C-3 substituted indole derivatives

Beata Jasiewicz[a*], Weronika Kozanecka-Okupnik[a], Michał Przygodzki[b], Beata Warżajtis[a], Urszula Rychlewska[a], Tomasz Pospieszny[a], Lucyna Mrówczyńska[b*]

[a]*Faculty of Chemistry, Adam Mickiewicz University, Uniwersytetu Poznańskiego 8, 61-614 Poznań, Poland*
[b]*Department of Cell Biology, Faculty of Biology, Adam Mickiewicz University, Uniwersytetu Poznańskiego 6, 61-614 Poznań, Poland*

TABLE OF CONTENTS

Spectroscopic study of indole derivatives……………………………………………………...S2
^{1}H and ^{13}C NMR spectra of *N*-acetyl-indole-3-carbinol **3** S5
^{1}H and ^{13}C NMR spectra of 3-Propoxy I3C **6** S6
^{1}H and ^{13}C NMR spectra of 3-Isopropoxy I3C **7** S7
^{1}H and ^{13}C NMR spectra of (*1H*-indol-2-yl)methylpyrrolidine-1-carbodithioate **12**.............. S8
Table S1 Crystal data and data collection, and refinement details... S9
Figure S8. Molecular models of gramine **1**, and its derivatives **3-13** calculated by the PM5 method…………………………………………………………...………………………S10
Table S2. Heat of formation (HOF) of gramine **1**, and its derivatives **11–15**……………...S12

* Corresponding author. Tel.: +48-61-8291354; fax.: +48-61-8291505. *E-mail*: beatakoz@amu.edu.pl (Beata Jasiewicz), lumro@amu.edu.pl (LucynaMrówczyńska)

*N,O-diacetyl-indole-3-carbinol **(2)***

Solid, (162.5 mg, 70%); mp 89-90 °C (lit.[1] 88-90 °C); ^{1}H NMR (300 MHz, $CDCl_3$): δ 8.45-8.42 (d, *J* = 8 Hz, 1H), 7.60-7.30 (m, 4H, indole ring), 5.27 (s, 2H, $-CH_2O-$), 2.63 (s, 3H, CH_3CO-), 2.09 (s, 3H, CH_3COO^-); ^{13}C NMR (75 MHz, $CDCl_3$): δ 171.0, 168.5, 135.9, 129.2, 125.6, 124.9, 123.8, 119.0, 117.3, 116.7, 57.8, 23.9, 21.0; IR (KBr): 3115, 1732, 1705, 1607, 1457, 1342, 1331, 1249 cm^{-1}; EI-MS (*m*/*z*, % int.): 231 (M^+, 100). Elem. Anal. (calcd., found for $C_{13}H_{14}NO_3$): C (67.23, 67.85), H (6.08, 6.17), N (6.03, 6.01).

*N-acetyl-indole-3-carbinol (**3**)*

Solid, (37.8 mg, 40%); m.p.127-129 °C; ^{1}H NMR (300 MHz, $CDCl_3$): δ = 7.64-7.25 (m, 4H, indole ring), 4.87 (s, 2H, $-CH_2OH$), 2.60 (s, 3H, CH_3CO-); ^{13}C NMR (75 MHz, $CDCl_3$): δ 168.0, 136.2, 129.1, 125.6, 123.7, 122.8, 119.1, 116.7, 111.8, 57.3, 23.9; FT-IR (KBr): 3500-3000, 1687, 1606, 1451 cm^{-1}; EI MS (*m*/*z*, % int.): 189 (M^+, 75). Elem. Anal. (calcd., found for $C_{11}H_{11}NO_2$): C (69.83, 69.93), H (5.85, 5.72), N (7.40, 7.31).

*3-Methoxy-indole-3-carbinol (**4**)*

Solid, (101 mg, 63%); m.p. 95-96 °C (lit.[1] 93-94 °C); ^{1}H NMR (300 MHz, DMSO-*d*$_6$): δ = 10.40 (s, 1H, NH), 7.72-6.73 (m, 5H, indole ring), 4.62 (s, 2H, $-O-CH_2-$), 3.29-3.25 (m, 3H, -O-CH_3); ^{13}C NMR (75 MHz, DMSO-*d*$_6$): δ 136.3, 127.1, 124.7, 123.8, 121.7, 121.2, 118.8, 111.5, 63.9, 57.3. EI MS (*m*/*z*, % int.): 161 (M^+, 78). Elem. Anal. (calcd., found for $C_{10}H_{11}NO$): C (74.51, 74.47), H (6.88, 6.57), N (8.69, 8.43).

*3-Ethoxy-indole-3-carbinol **(5)***

Solid, (123 mg, 70%); m.p. 64-66 °C (lit.[1] 63-65 °C); ^{1}H NMR (300 MHz, DMSO-*d*$_6$): δ = 9.93 (s, 1H, NH), 7.58-6.97 (5H, indole ring), 4.72 (s, 2H, indole ring-CH_2O-), 3.58 (m, 2H, -O-CH_2-), 1.24 (t, 3H, $-CH_3$). ^{13}C NMR (75 MHz, DMSO-*d*$_6$): δ = 136.3, 127.0, 124.6, 123.3, 121.9, 121.1, 118.7, 111.4, 63.9, 63.8, 15.2. EI-MS (*m*/*z*, % int.): 175 (M^+, 63). Elem. Anal. (calcd., found for $C_{11}H_{14}NO$): C (74.97, 74.85), H (8.01, 8.17), N (7.95, 7.83).

*3-Propoxy-indole-3-carbinol (**6**)*

Brown oil, (151 mg, 80%); ^{1}H NMR (300 MHz, $CDCl_3$): δ = 8.11 (bs, 1H, NH), 7.72-7.10 (m, 5H, indole ring), 4.72 (s, 2H, $-CH_2$-O), 3.48 (t, *J* = 6.8 Hz, 2H), 1.69-1.58 (m, 2H, $-CH_2-$), 0.95-0.90 (t, *J* = 7.4 Hz, 3H); ^{13}C NMR (75 MHz, $CDCl_3$): δ = 136.4, 127.2, 123.4, 122.2, 119.7, 119.3, 113.7, 111.1, 71.6, 64.9, 23.0, 10.7; EI MS (*m*/*z*, % int.): 189 (M^+, 40). Elem. Anal. (calcd., found for $C_{12}H_{15}NO$): C (76.16, 76.07), H (7.99, 7.82), N (7.40, 7.25).

*3-Isopropoxy-indole3-carbinol (**7**)*

Brown oil, (142 mg, 75%); ^{1}H NMR (300 MHz, $CDCl_3$): δ = 8.07 (bs, 1H, NH), 7.71-7.10 (m, 5H, indole ring), 4.72 (s, 2H, $-CH_2-O$), 3.77 (q, J = 6.1, 1H), 1.23 (d, J = 6.1 Hz, 6H); ^{13}C NMR (75 MHz, $CDCl_3$): δ = 136.4, 127.1, 123.2, 122.1, 119.7, 119.3, 113.9, 111.0, 70.2, 62.1, 22.2; EI MS (*m*/*z*, % int.): 189 (M^+, 30). Elem. Anal. (calcd., found for $C_{12}H_{15}NO$): C (76.16, 76.24), H (7.99, 8.06), N (7.40, 7.51).

*3-Butoxy-indole-3-carbinol (**8**)*

Brown oil, (112 mg, 55%); ^{1}H NMR (300 MHz, $CDCl_3$): δ = 8.10 (s, 1H, NH), 7.74-7.11 (m, 5H, indole ring), 4.71 (s, 2H, $-CH_2-O$), 3.60-3.52 (m, 2H, $-O-CH_2-$), 1.70-0.90 (m, 7H); ^{13}C NMR (75 MHz, $CDCl_3$): δ = 136.4, 127.2, 123.3, 122.1, 119.7, 119.3, 113.8, 111.1, 71.3, 65.9, 22.9, 21.5, 13.7; EI MS (*m*/*z*, % int.): 203 (M^+, 10). Elem. Anal. (calcd., found for $C_{13}H_{17}NO$): C (76.81, 76.92), H (8.43, 8.39), N (6.89, 6.94).

*3-Pentoxy-indole3-carbinol (**9**)*

Brown oil, (76 mg, 35%); ^{1}H NMR (300 MHz, $CDCl_3$): δ = 8.10-7.05 (m, 6H, NH, indole ring), 5.37 (s, 2H, $-CH_2-O$), 3.45-3.43 (m, 2H, $-O-CH_2-$), 1.70-1.23 (m, 6H), 0.92-0.88 (m, 3H, $-CH_3$); ^{13}C NMR (75 MHz, $CDCl_3$): δ = 136.7, 127.4, 123.2, 122.3, 119.5, 119.1, 113.8, 111.2, 71.5, 65.7, 22.7, 20.5, 12.7, 10.5; EI MS (*m*/*z*, % int.): 217 (M^+, 15). Elem. Anal. (calcd., found for $C_{14}H_{19}NO$): C (77.38, 77.15), H (8.81, 8.73), N (6.45, 6.32).

*3-Isopentoxy-indole3-carbinol (**10**)*

Brown oil, (137 mg, 63%); ^{1}H NMR (300 MHz, $CDCl_3$): δ = 8.05-7.00 (m, 6H, NH, indole ring), 5.36 (s, 2H, $-CH_2-O$), 3.48-3.45 (m, 2H, $-O-CH_2-$), 1.74-1.43 (m, 3H), 0.86-0.84 (m, 6H, $-CH_3$); ^{13}C NMR (75 MHz, $CDCl_3$): δ = 136.8, 127.4, 123.1, 122.4, 119.6, 119.1, 113.6, 111.2, 71.6, 65.5, 22.8, 21.5, 15.7, 15.2; EI MS (*m*/*z*, % int.): 217 (M^+, 6). Elem. Anal. (calcd., found for $C_{14}H_{19}NO$): C (77.38, 77.45), H (8.81, 8.94), N (6.45, 6.52).

*3,3'-Diindolylmethane DIM (**11**)*

Solid, (25 mg, 20%); m.p. 166-168 °C (lit.[2] 167-169 °C); ^{1}H NMR (300 MHz, $CDCl_3$): δ = 10.5 (bs, 2H), 7.00-8.05 (m, 12H, indole rings), 4.64 (s, 2H, $-CH_2-$); ^{13}C NMR (101 MHz, $CDCl_3$): δ = 137.2, 127.8, 122.8, 121.9, 119.6, 115.9, 110.3, 75.7, 21.2; EI MS (*m*/*z*, % int.): 246 (M^+, 100).

*(1H-indol-2-yl)methylpyrrolidine-1-carbodithioate (**12**)*

Brown oil, (69 mg, 25%); ^{1}H NMR (403 MHz, $CDCl_3$): δ = 8.33 (s, 1H, NH), 7.72-7.11 (m, 5H, indole ring), 4.77 (s, 2H, $-CH_2-S$), 3.97-3.86 (m, 2H, $N-CH_2-$), 3.56-3.53 (m, 2H, $N-CH_2-$), 2.07-1.84 (m, 4H, $-CH_2-CH_2$); ^{13}C NMR (101 MHz, $CDCl_3$): δ = 192.9, 136.1, 126.8, 124.0,

122.2, 119.6, 118.9, 111.3, 109.9, 54.7, 50.5, 32.8, 25.9, 24.2; FT-IR (KBr): 3405, 3054-2870, 1617, 1547, 1436, 1338, 1248, 1181 cm^{-1}; EI MS (*m/z*, % int.): 276 (M^+, 8); Elem. Anal. (calcd., found for $C_{14}H_{16}N_2S_2$): C (60.83, 60.79), H (5.83, 5.74), N (10.13, 10.17), S (23.20, 23.18).

3-Imidazol-1-ylmethyl-indole ***(13)***

Solid, (187 mg, 95%); m.p. 170-171 °C (lit.[3] 172-174 °C); ^{1}H NMR (403 MHz, $CDCl_3$): δ = 12.13 (bs, 1H, NH), 7.72 (s, 1H, N-CH=N), 7.60-7.02 (m, 5H, indole ring), 7.12 (s, 2H, N-CH=CH-N), 5.31 (s, 2H, CH_2-N); ^{13}C NMR (101 MHz, $CDCl_3$): δ = 136.7, 135.2, 128.5, 126.1, 124.2, 122.4, 121.8, 119.9, 119.4, 118.0, 42.8; FT-IR (KBr): 3124-2615, 1542, 1448, 1326, 1262 cm^{-1}; EI MS (*m/z*, % int.): 197 (M^+, 20); Elem. Anal. (calcd., found for $C_{12}H_{11}N_3$): C (73.07, 73.14), H (5.62, 5.59), N (21.30, 21.27).

*N-Indol-3-ylmethyl-phtalimide (**14**)*

Solid, (218 mg, 79%); m.p. 180-182 °C (lit.[4] 181-183 °C); ^{1}H NMR (403 MHz, $CDCl_3$): δ = 8.09 (bs, 1H, NH), 7.95 (s, 1H), 7.78 (d, *J* = 3.0, 2H), 7.64 (s, 2H), 7.39 (s, 1H), 7.33 (d, *J* = 7.0 Hz, 1H), 7.15 (s, 2H), 5.03 (s, 2H); ^{13}C NMR (101 MHz, $CDCl_3$): δ = 168.2, 135.9, 133.8, 132.2, 126.4, 125.1, 123.1, 122.4, 120., 119.4, 111.4, 111.2, 32.6; FT-IR (KBr): 3389, 3108, 3069, 2943, 1764, 1696 cm^{-1}; EI MS (*m/z*, % int.): 276 (M^+, 75); Elem. Anal. (calcd., found for $C_{17}H_{12}N_2O_2$): C (73.90, 73.76), H (4.38, 4.30), N (10.14, 10.19).

*N-(N'-acetylindol-3-yl)methyl-phtalimide (**15**)*

Solid, (92 mg, 80%); m.p. 196-198 °C (lit.[5] 200-200.5 °C); ^{1}H NMR (403 MHz, $CDCl_3$): δ = 8.40 (s, 1H, NH), 7.86 (ddd, *J* = 7.3, 1.7, 0.8 Hz, 1H), 7.84-7.79 (m, 2H, ArH), 7.73-7.67 (m, 2H, ArH), 7.6 (s, 1H), 7.44-7.29 (m, 2H), 4.97 (s, 2H), 2.64 (s, 3H); ^{13}C NMR (101 MHz, $CDCl_3$): δ = 168.6, 168.0, 135.7, 134.1, 132.0, 129.1, 125.7, 125.5, 123.9, 123.3, 119.4, 117.3, 116.6, 32.2, 24.0; FT-IR (KBr): 3459, 3125, 3051, 1768, 1712, 1610, 1453 cm^{-1}; EI MS (*m/z*, % int.): 318 (M^+, 25), 276 (25), 130 (50). Elem. Anal. (calcd., found for $C_{19}H_{14}N_2O_3$): C (71.69, 71.60), H (4.43, 4.51), N (8.80, 8.76).

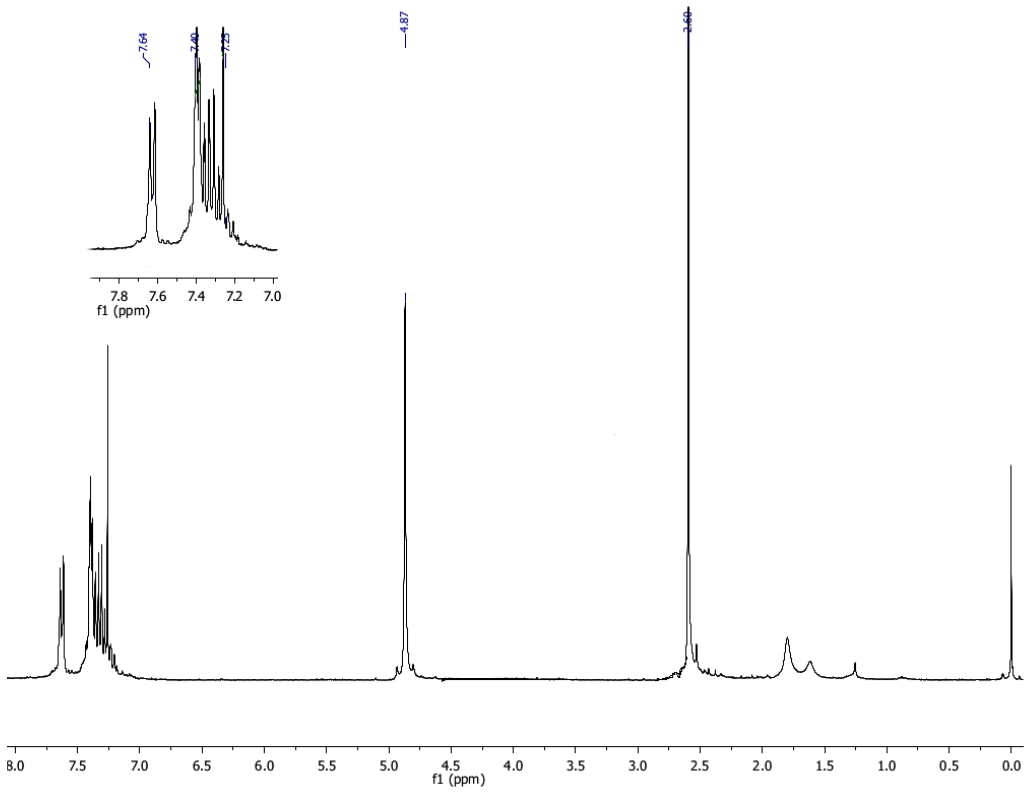

Figure S1a. ^{1}H NMR spectrum of *N*-acetyl-indole-3-carbinol **3**.

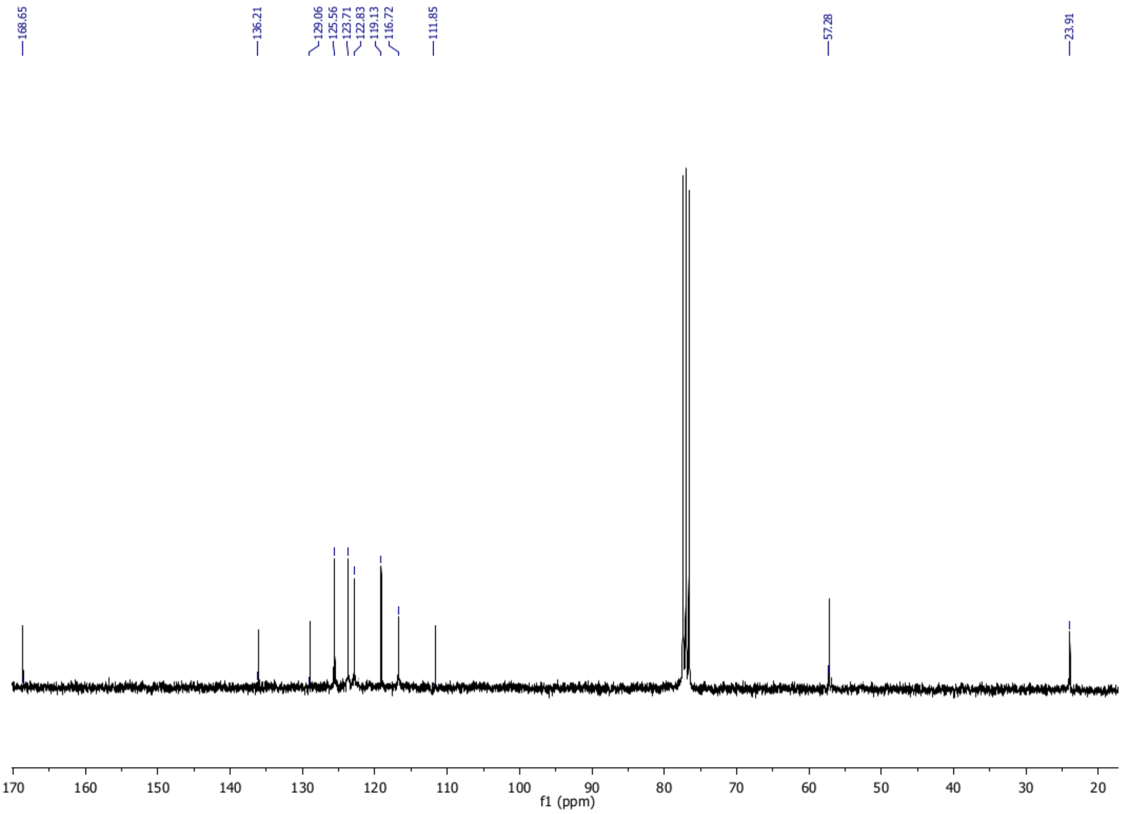

Figure S1b. ^{13}C NMR spectrum of *N*-acetyl-indole-3-carbinol **3**.

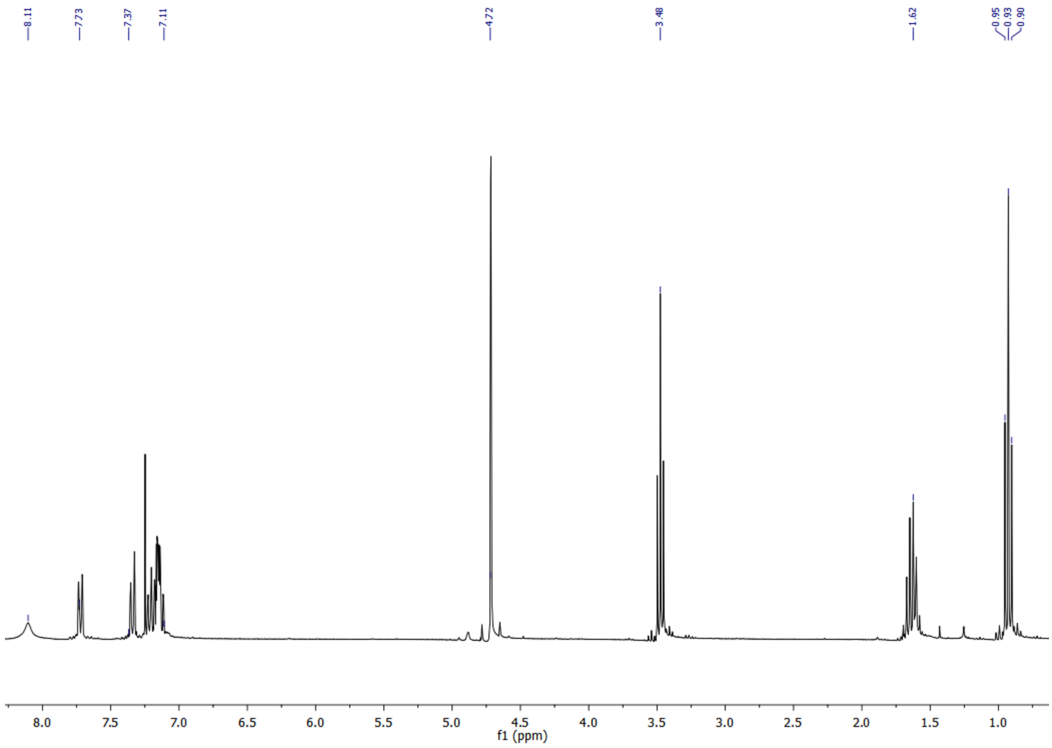

Figure S2a. ^{1}H NMR spectrum of 3-Propoxy I3C **6**.

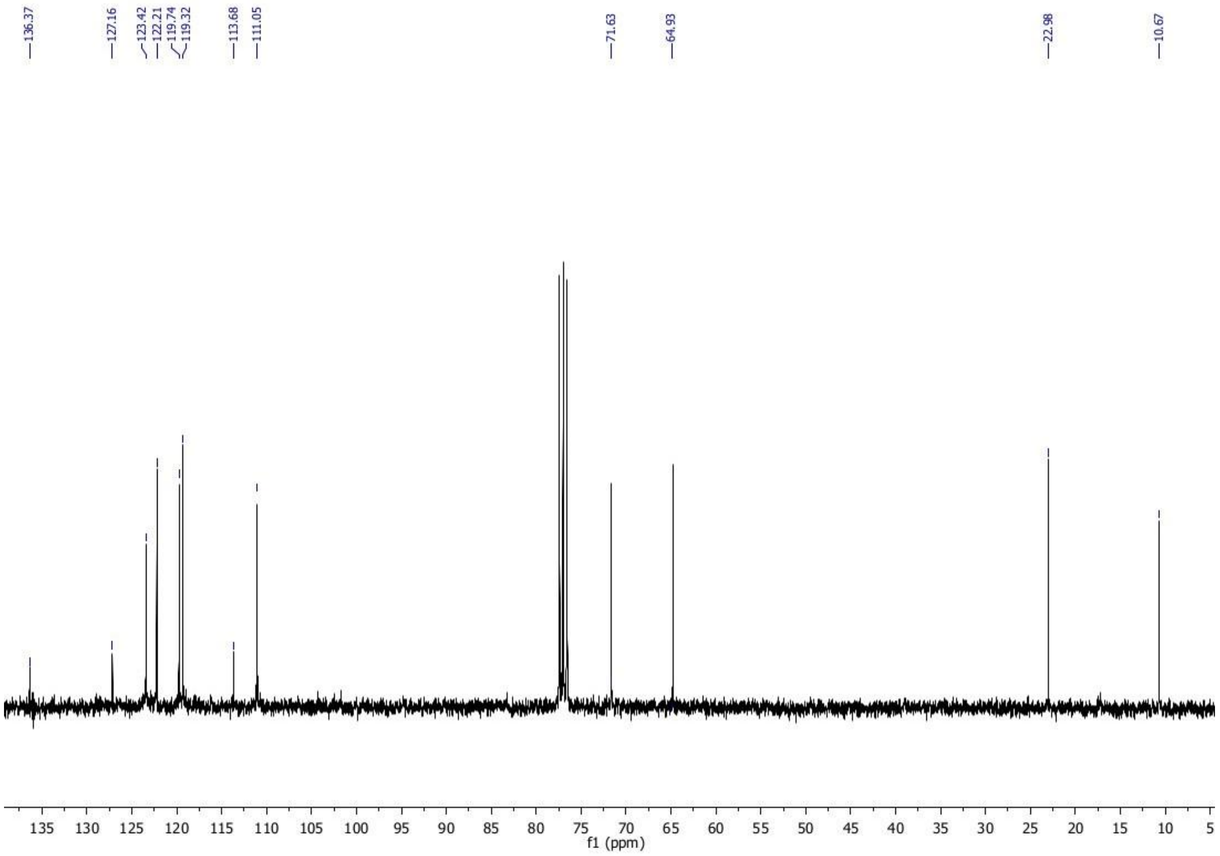

Figure S2b. ^{13}C NMR spectrum of 3-Propoxy I3C **6**.

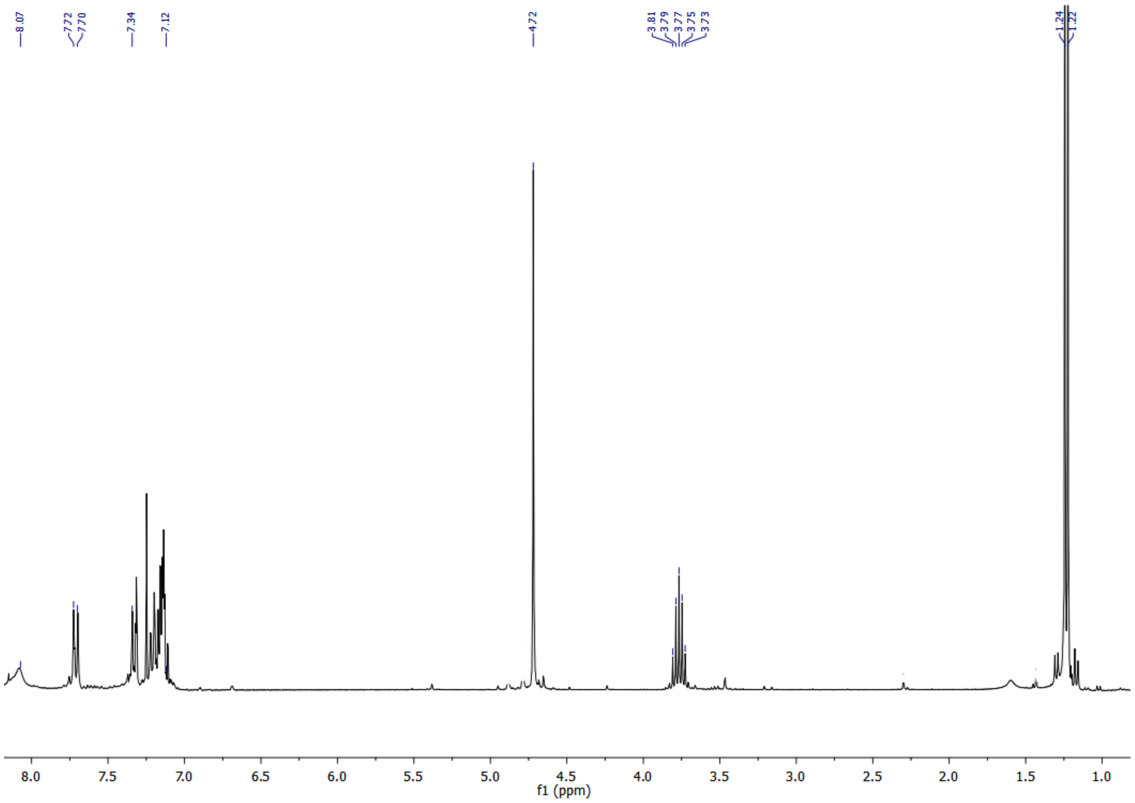

Figure S3a. ^{1}H NMR spectrum of 3-Isopropoxy I3C **7**.

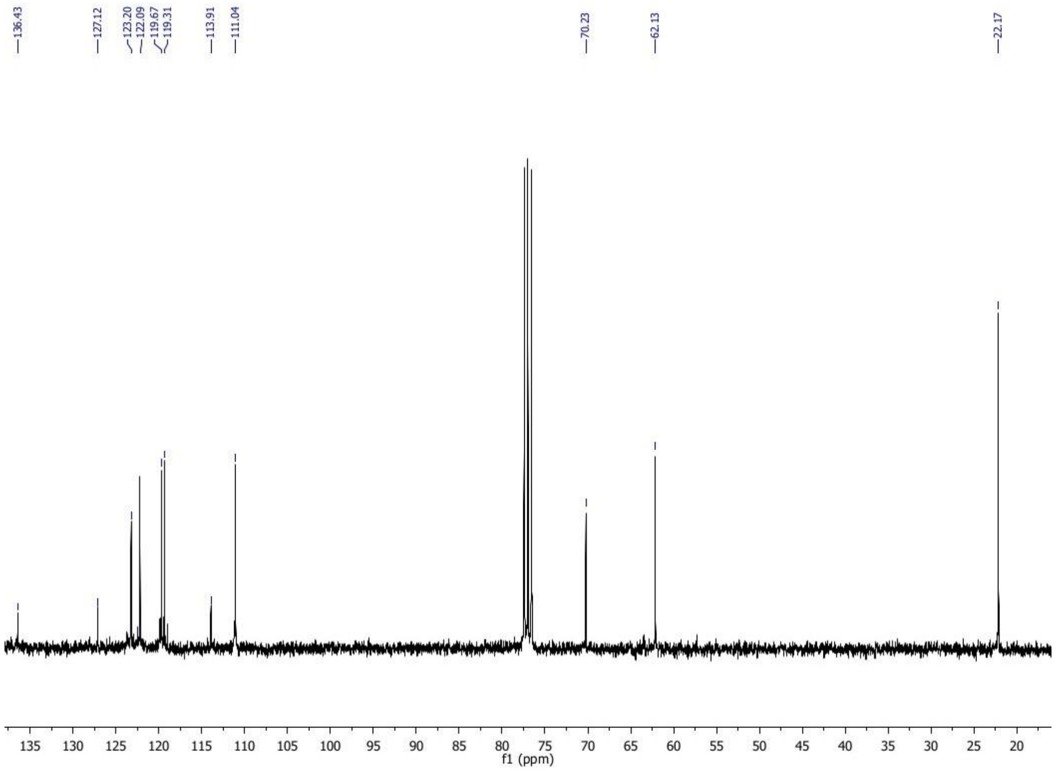

Figure S3b. ^{13}C NMR spectrum of 3-Isopropoxy I3C **7**.

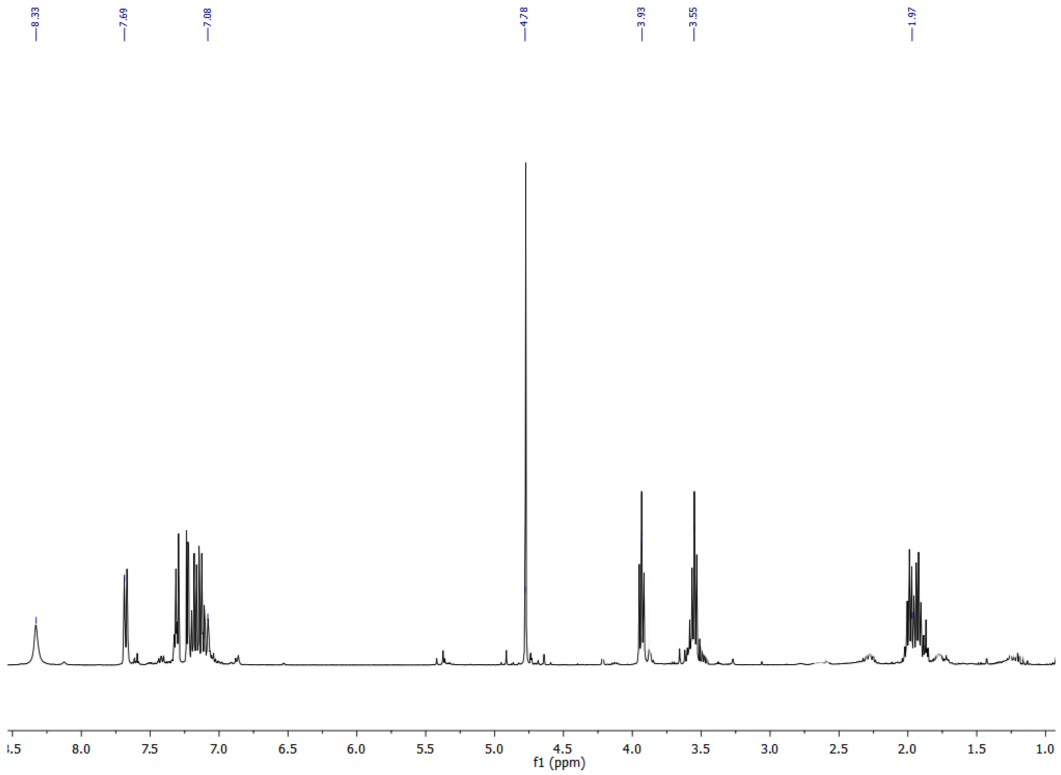

Figure S4a. ^{1}H NMR spectrum of (*1H*-indol-2-yl)methylpyrrolidine-1-carbodithioate **12**.

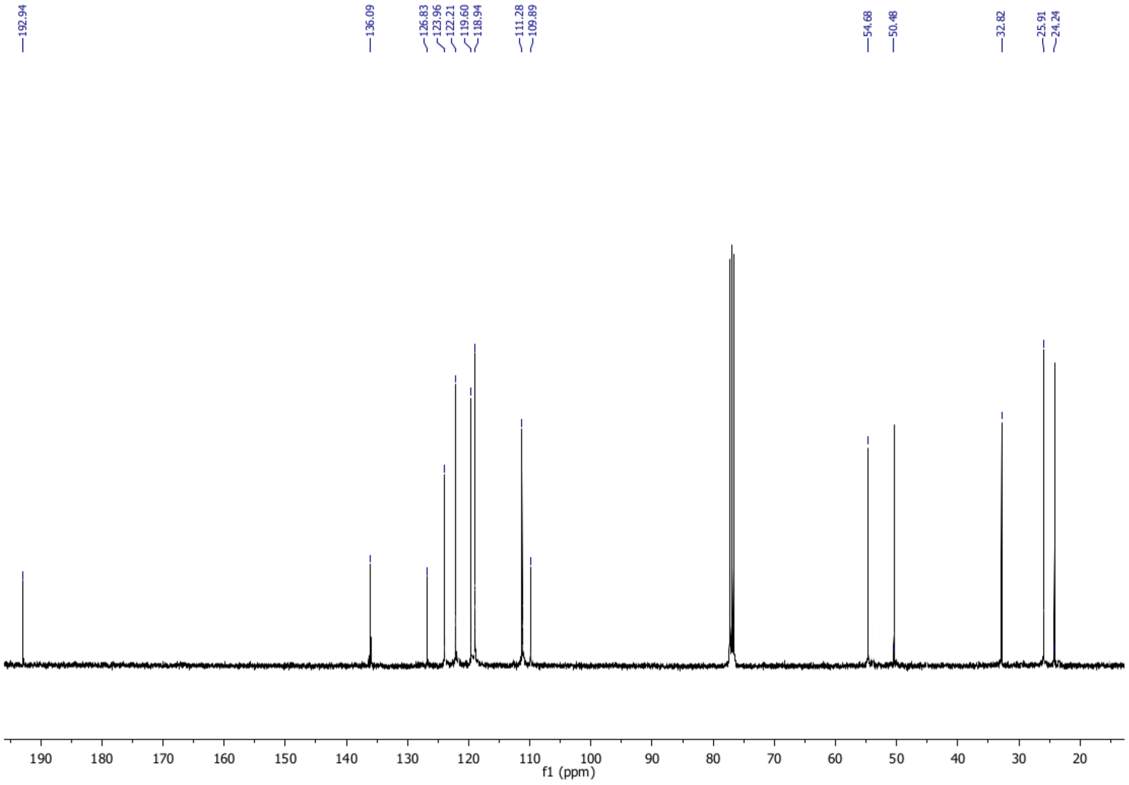

Figure S4b. ^{13}C NMR spectrum of (*1H*-indol-2-yl)methylpyrrolidine-1-carbodithioate **12**.

Table S1. Crystal data and data collection, and refinement details

	(**2**)	(**14**)	(**15**)
Crystal data			
Chemical formula	$C_{13}H_{13}NO_3$	$C_{17}H_{12}N_2O_2$	$C_{19}H_{14}N_2O_3$
M_r	231.24	276.29	318.32
Crystal system, space group	Monoclinic, *C*2/*c*	Orthorhombic, $Pna2_1$	Triclinic, $P\bar{1}$
Temperature (K)	295		
a, *b*, *c* (Å)	19.2159 (4), 7.4704 (2), 16.7645 (3)	6.9761 (1), 17.7481 (1), 10.9011 (1)	4.7470 (2), 12.8370 (8), 13.4424 (7)
α, β, γ (°)	90, 100.070 (2), 90	90, 90, 90	69.868 (5), 81.294 (4), 81.284 (5)
V (Å^3)	2369.48 (9)	1349.69 (2)	755.86 (7)
Z	8	4	2
μ (mm^{-1})	0.76	0.74	0.79
Crystal size (mm)	0.50 × 0.23 × 0.02	0.45 × 0.30 × 0.10	0.55 × 0.05 × 0.02
Data collection			
Diffractometer	SuperNova, Single source at offset, Atlas		
T_{min}, T_{max}	0.608, 1.000	0.714, 1.000	0.852, 1.000
No. of measured, independent and observed [$I > 2\sigma(I)$] reflections	12989, 2084, 1922	60142, 2385, 2370	7543, 2668, 2426
R_{int}	0.022	0.041	0.017
$(\sin \theta/\lambda)_{max}$ (Å^{-1})	0.595	0.595	0.595
Refinement			
$R[F^2 > 2\sigma(F^2)]$, $wR(F^2)$, S	0.053, 0.166, 1.09	0.029, 0.073, 1.07	0.037, 0.104, 1.04
No. of reflections	2084	2385	2668
No. of parameters	156	195	218
No. of restraints	0	14	0
$\Delta\rho_{max}$, $\Delta\rho_{min}$ (e Å^{-3})	0.42, -0.27	0.12, -0.13	0.14, -0.19
Absolute structure parameter	–	0.08 (5)	–

Figure S8. Molecular models of gramine (**1**), and its derivatives **3-13** calculated by the PM5 method (WinMopac 2003)[6].

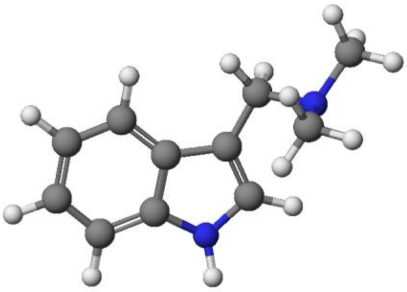

1

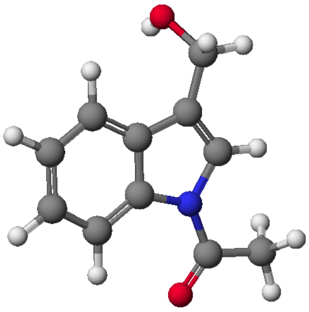

3

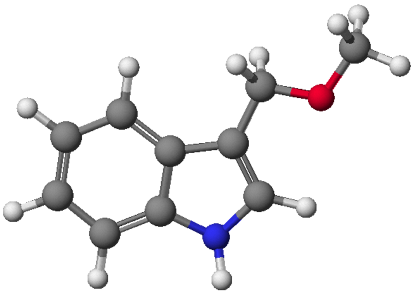

4

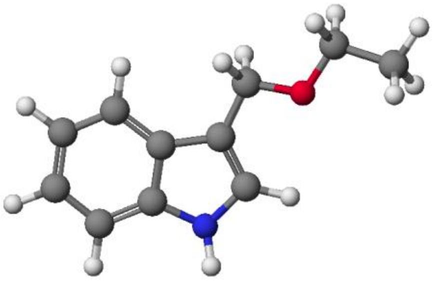

5

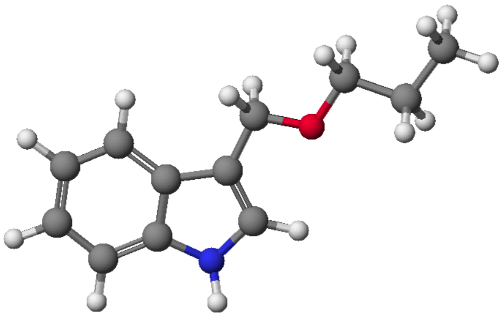

6

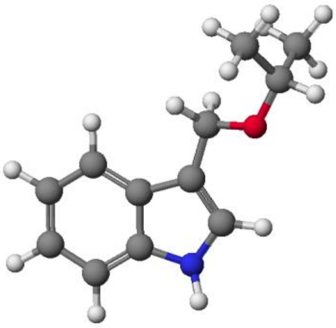

7

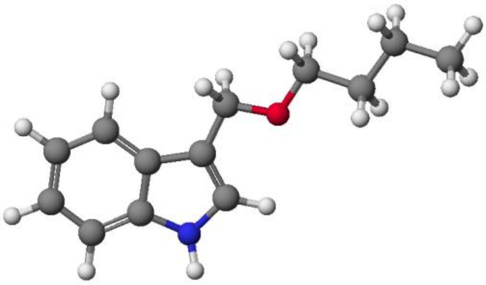

8

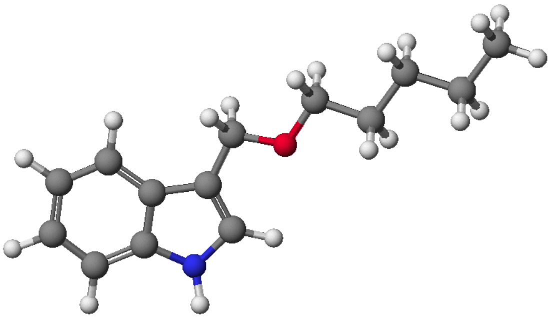

9

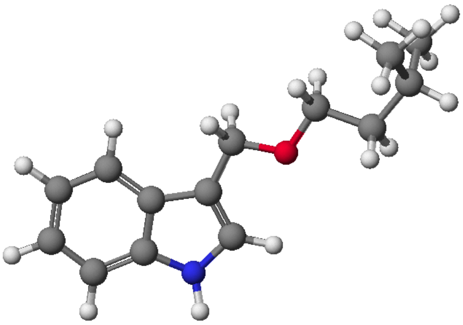

10

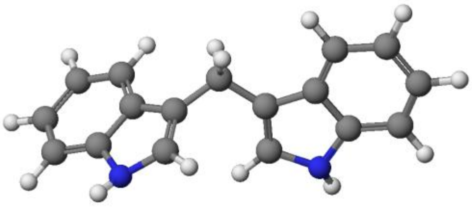

11

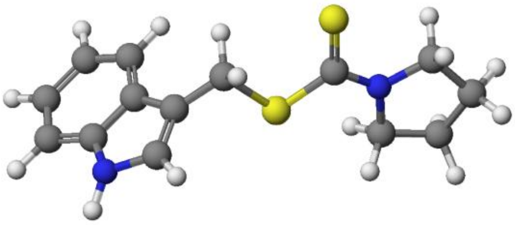

12

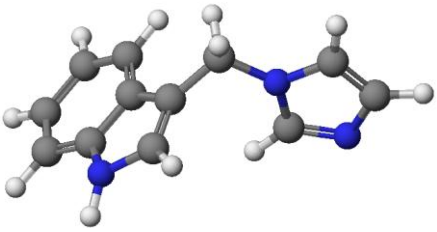

13

Table S2. Heat of formation (HOF) [kcal/mol] of gramine (**1**), and its derivatives (**11**–**15**).

Number of compound	HOF [kcal/mol]
**1**	47.9987
**2**	–87.7940
**3**	–44.3566
**4**	5.3853
**5**	–0.5144
**6**	–5.9727
**7**	–9.8850
**8**	–11.4646
**9**	–17.0637
**10**	–16.6643
**11**	85.2579
**12**	58.4619
**13**	84.3824
**14**	–38.3733
**15**	–45.2249

References

1. Jump, S. M. et al. N-Alkoxy derivatization of indole-3-carbinol increases the efficacy of the G1 cell cycle arrest and of I3C-specific regulation of cell cycle gene transcription and activity in human breast cancer cells. *Biochemical pharmacology* **75**, 713-724 (2008).
2. Abe, T et al. One-Pot Construction of 3,30-Bisindolylmethanes through Bartoli Indole Synthesis. *Org. Lett*, **15**, 3622-3625 (2013).
3. Le Borgne, M. et al. Synthesis and *In Vitro* Evaluation of 3-(1-Azolylmethy1)- 1*H*-indoles and 3(1-Azolyl-l-phenylmethyl)-1*H*-indoleass Inhibitors of P450 arom. *Arch. Pharm. Pharm. Med. Chem.* 330, 141-145 (1997),

4.Iwao, M., Motoi, O. Methodology for the efficient synthesis of 3,4-differentially substituted indoles. Fluoride ion-induced elimination-addition reaction of 1-triisopropylsilylgramine methiodides, *Tetrahedron Lett.* **36**, 5929-5932 (1995).

5. Takechi, H., Machida, M., Kanaoka, Y. Intramolecular photoreactions of phthalimide-alkene systems. O Formation of *N*-(ω-indol-3-ylalkyl)phthalimides. *Chem. Pharm. Bull.* **36,** 2853-2863 (1988).
6. CAChe 5.04 User Guide, Fujitsu Chiba, Japan (2003).
